# Supplementary material for: Molecular epidemiology and antimicrobial susceptibility of diarrheagenic Escherichia coli isolated from children under age five with and without diarrhea in Central Ethiopia
Source: PLoS One. 2023 Jul 14;18(7):e0288517. doi: 10.1371/journal.pone.0288517 (PMC10348587; doi:10.1371/journal.pone.0288517)
Supplement: S1 File — (DOCX) [file pone.0288517.s001.docx]

## Questionnaire (English version)

**Questionnaire for assessment of factors associated with diarrhea in Addis Ababa and Debre Berhan, Ethiopia.**

1. **Patient information**
   1. Name of health facility _________________________
   2. Identification number (card number) _________________
   3. Address________________________
2. **Socio-demographic information for under-five children**

| S.No | Questions (for child and caregiver) | Response |
| --- | --- | --- |
|  | Age of the child | __________ (in month) or _______ (in year) |
|  | Sex of the child | □ Male □ Female |
|  | Relation of the respondent to the child? | □ Mother □ Guardian (caregiver) □ Other (specify) |
|  | Age of the mother or guardian (caregiver) | __________ |
|  | Sex of the guardian (if not mother) | □ Male □ Female |
|  | How many person are in the household? | __________ |
|  | How many children are in the family? | ________ |
|  | Birth order | ________ |
|  | Educational level of the mother or guardian | □ Illiterate □ Primary □ Junior secondary  □ Preparatory □ College or University |
|  | Educational level of the father | □ Illiterate □ Primary □ Junior secondary  □ Preparatory □ College or university |
|  | Marital status of the mother or guardian | □ Single □ Married □ Other (specify) |
|  | Ethnic groups | □ Amhara □ Oromo □ Tigray  □ Other (specify) |
|  | Occupation (Mother) | □ Employed □ Self-employed □ Other (specify) |
|  | Occupation (Father) | □ Employed □ Self-employed □ Other (specify) |
|  | Family income per month (in birr) | __________ |

1. **Clinical features**

|  | Time of illness started | __________ |
| --- | --- | --- |
|  | Duration of diarrhea in days | __________ |
|  | Stool frequency per day | __________ |
|  | Types of diarrhea | □ Watery □ Mucoid □ Bloody □ Loose |
|  | Child dehydration status | □ Mild □ Moderate □ Severe □ None |
|  | Other clinical feature |  |
|  | Fever | □ Yes □ No |
|  | Vomiting | □ Yes □ No |
|  | Nausea | □ Yes □ No |
|  | Increased thirst | □ Yes □ No |
|  | Abdominal distension | □ Yes □ No |
|  | Other (specify) | __________ |
|  | Previous treatment | □ Yes □ No |
|  | If yes when? | _________ |

1. **Behavioural and health related factors related to under-five children**

|  | How the child is cared daily at home? | □ With house worker □ With mother  □ In day care □ Other (specify)___ |
| --- | --- | --- |
|  | Feeding practice | □ Exclusive breast milk □ Breast milk & solid food  □ Solid food only □ Formula Milk |
|  | Duration of breastfeeding | □ < 1year □ 1-2 years □ > 2years |
|  | Beginning age of supplementary feeding | □ < 6 moths □ 6-12 moths □ > 12 months |
|  | What method do you use to feed your child? | □ Hand □ Cup or spoon □ Bottle |
|  | When do you wash your hand? | □ Before food preparation □ Before and after feeding □ After latrine □ After cleaning child’s bottom |
|  | What do you use for hand washing? | □ Soaps □ Water only □ Others |
|  | Do your child eat by himself/herself? | □ Yes □ No |
|  | Do your child wash his/her hands before feeding? | □ Yes □ No |
|  | How the child dispose his/her feces? | □ With diaper □ Latrine □ Ground  □ On baby potty (popo) □ Under clothes |
|  | Where do you dispose your child’s stool? | □ Child use latrine □ Put into latrine  □ Thrown in garbage □ Buried □ Left on the ground |
|  | Where do you dispose water used for washing your child’s stool? | □ Put in latrine □ Thrown on the ground  □ Thrown in the garbage □ No water used |
|  | What vaccines your child has taken? | □ Measles vaccine □ Rota vaccine  □ Other (specify) |
|  | Nutritional status of the child | □ Under nutrition □ Normal |
|  | Is there any family member who travel abroad? | □ Yes □ No □ If yes, when? ________ |

1. **Environmental exposure variables**

|  | House ownership | □ Private □ Rented □ Other (specify) |
| --- | --- | --- |
|  | Is a separate kitchen available? | □ Yes □ No |
|  | Owner ship of latrine | □ Shared □ Private □ Public |
|  | What types of toilet does the household mainly use? | □ Pit latrine with slab □ Pit latrine without a slab or pour-flush latrine □ Open field □ Other (specify)_____ |
|  | Does hand washing facility is available near to latrine? | □ Yes □ No |
|  | How often is the latrine cleaned? | □ Every time it is spoiled □ Every day □ 1-2 times a week □ Not cleaned |
|  | Where is your refuse container (bin) located? | □ Kept indoors □ Outdoors □ Do not own a bin |
|  | Does your bin have a properly fitting lid? | □ Yes □ No |
|  | If your waste is not collected, how do you dispose of your waste? | □ Burn □ Burry □ Dump □ Other (specify)____ |
|  | How often is your waste/refuse collected by local authority? | □ Daily □ Weekly □ Monthly □ Never □ Other (specify)_____ |
|  | Are there any domestic animals in the compound? | □ Yes □ No |
|  | What types of animals are in the house or the yard? | □ Cattle □ Goats □ Dog □ Chicken □ Other specify |
|  | Where do you mainly get your drinking water from? | □ Tape water □ Packed water □ Protected spring/well water □ Unprotected spring/well water □ Other __ |
|  | Time to obtain drinking water (shift) | □ No shift (daily) □ Shift □ if shift, how long? ____ |
|  | What treatment of water do you use? | □ Filtering □ Boiling □ None □ Other (specify)___ |
|  | What kind of utensils do you use for storing water? | □ Storage containers without lid  □ Storage containers with lid |
|  | Do you always clean and empty the storage container before replacing with fresh water? | □ Yes □ No |

**Data collector name_______________ Date __________ Signature___________**

**ቃለ-መጥይቅ**

ዕድሚያቸው ከ5 ዓመት በታች በሆኑ ህጻናት ላይ ለሚከሰተው የተቅማጥ በሽታ ተያያዥነት ያላቸውን ነገሮች ለዳሰስ የተዘጋጀ መጥይቅ

**ሀ. የህፃናት (የታካሚው) መረጃ**

1. የህክምና አገልግሎት መስጫ ስም _________________
2. የህመምተኛ መለያ ቁጥር (ካርድ ቁጥር) _________________
3. አድራሻ _________________

**ለ. ማህበራዊና የወሳኝ ኩነቶች መረጃ**

| ተ.ቁ. | መጥይቆች(ለህጻኑና አሳዳጊ) | ምላሽ |
| --- | --- | --- |
|  | የህፃኑ/ኗ ዕድሜ | _______ |
|  | የህፃኑ/ኗ ፆታ | □ ወንድ □ ሴት |
|  | ከህፃኑ/ኗ ጋር ያለው ዝምድና | □ ወላጅ እናት □ አሳዳጊ □ ሌላ (ይጠቀስ) |
|  | የህፃኑ/ኗ እናት/አሳዳጊ ዕድሜ | __________ |
|  | የህፃኑ/ኗ አሳዳጊ ጾታ (ከእናት ውጭ ከሆነ) | □ ወንድ □ ሴት |
|  | የቤተሰብ ብዛት ስንት ነው? |  |
|  | በቤት ውስጥ ምን ያህል ህጻናት አሉ? | ________ |
|  | ስንተኛ ልጅ ነው? |  |
|  | የህጻኑ አሳዳጊ የትምህርት ደርጃ | □ ያልተማረ □ የመጀመሪያ ደረጃ  □ መለስተኛና ሁለተኛ ደረጃ □ መሰናዶ  □ ኮሌጅ ወይም ዩኒቨርስቲ |
|  | የአባት የትምህርት ደረጃ | □ መለስተኛና ሁለተኛ ደረጃ □ መሰናዶ  □ ኮሌጅ ወይም ዩኒቨርስቲ |
|  | የህጻኑ አሳዳጊ የጋብቻ ሁኔታ | □ ያላገባ □ ያገባ □ ሌላ (ይገለጽ) |
|  | የስራ ሁኔታ (የእናት) | □ ተቀጣሪ □ የራስ ተቀጣሪ  □ ሌላ (ይገለጽ) |
|  | የስራ ሁኔታ (የአባት) | □ ተቀጣሪ □ የራስ ተቀጣሪ  □ ሌላ (ይገለጽ) |
|  | የቤተሰብ ወርሃዊ ገቢ (በብር) |  |
|  | ባለፈው ሳምንት ውስጥ ጥሬ አትክልት፣ፍራፍሬና ስጋ ልጆትን አብልተዉታል? | □ አዎ □ አላበላሁም |

**ሐ. ከህጻኑ በሽታ ጋር የሚያያዙ ምልክቶችና ተያያዥ ነገሮች**

| ተ.ቁ. | መጠይቆች | ምላሽ |
| --- | --- | --- |
|  | ህመሙ የጀመረበት ጊዜ | ___________ |
|  | ተቅማጡ የቆየበት ጊዜ (በቀን) | ___________ |
|  | የተቅማጥ ወይም የሰገራ ድግግሞሻ በቀን | ___________ |
|  | የተቅማጡ ዓይነት | □ ውሃማ □ ዝልግልግ □ ደም የቀላቀለ  □ የላላ (ቀጠን ያለ) |
|  | የህፃኑ/ኗ ውሃነስነት (ድርቀት) ሁኔታ | □ ቀላል □ መካከለኛ □ አስከፊ □ የለም |
|  | ሌሎች የታዩ ምልክቶች |  |
|  | ትኩሳት | □ አዎ □ የለም |
|  | ማስታወክ | □ አዎ □ የለም |
|  | ማቅለሽለሽ | □ አዎ □ የለም |
|  | ውሃ የመጥማት መጨመር | □ አዎ □ የለም |
|  | የሆድ መነፋት | □ አዎ □ የለም |
|  | ሌላ (ይገለጽ) | ____________ |
|  | ከዚህ በፊት የተወሰደ መድሃኒት | □ አዎ □ የለም |
|  | ከተወሰደ መቼ? | ____________ |

**መ. ከህጻኑ ጋር ተያያዥነት ያላቸው ባህሪያትና ጤና ነክ አጋላጭ ነገሮች**

|  | ህፃኑ/ኗ በቤት ውስጥ የሚከባከብበት ሁኔታ | □ በቤት ሰራተኛ □ በወላጅ እናት □ ህጻናት መቆያ □ ሌላ (ይጠቀስ) |
| --- | --- | --- |
|  | የአመጋገብ ሁኔታ | □ የእናት ጡት ብቻ □ የጡት ወተትና ተጨማሪ ምግብ ጋር □ ምግብ ብቻ □ የተዘጋጀ ወተት |
|  | የእናት ጡት የጠባበት ጊዜ | □ ከ1 ዓመት ያንሳል □ 1-2 ዓመት □ ከ2 ዓመት በላይ |
|  | ተጨማሪ ምግብ የተጀመረበት ጊዜ | □ ከ6 ወር በፊት □6-12 ወር □ከ12 ወር በላይ |
|  | ልጆን የሚመግቡበት መንገድ ምድን ነው? | □ በእጅ □ በሳህን ወይም በማንኪያ □ በጡጦ |
|  | እጆን መቼና ምን ጊዜ ይታጠባሉ? | □ ከምግብ ዝግጅት በፊት □ልጆን ከመመገቤ በፊትና ኋላ  □ ከሽንት ቤት በኋላ □ ልጁን ካጸዳሁ (ካካ) በኋላ |
|  | እጆን ለመታጠብ ምን ይጠቀማሉ? | □ ሳሙና □ ውሃ ብቻ □ ሌላ------ |
|  | ህጻኑ በራሱ ይመገባል? | □ ይመገባል □ አይመገብም |
|  | ህጻኑ እጁን/ጇን ከምግብ በፊት ይታጠባል? | □ ይታጠባል □ አይታጠብም |
|  | ህጻኑ እነዴት ይጸዳዳል? | □ በዳይፐር □ ሽንት ቤት □ ፖፖ □ ወለሉ ላይ  □ በልብስ ላይ □ ሌላ (ጥቀስ) ----- |
|  | የህኑን ሰገራ እንዴት ነው የሚያስወግዱት? | □ ህጻኑ ሽንት ቤት ይጠቀማል □ ሽንት ቤት  □ ቆሻሻ ማጠራቀሚ ላይ □ ውጭ (መስክ) ይጣላል |
|  | ህኑን ያጸዱበትን ዉሃ የሚያስወግዱበት የት ነው? | □ ሽንት ቤት □ መስክ □ ቆሻሻ ማጠራቀሚያ  □ በውሃ አይጸዳዳም |
|  | ህጻኑ የተከተበው የክትባት ዓይነት? | □ ኩፍኝ □ ሮታ □ ሌላ (ይጠቀስ) |
|  | ኑትሪሽናል (ውፍረቱ) ደረጃው | □ ዝቅተኛ □ ጤናማ □ ከፍተኛ |
|  | ባለፉት 2 ሳምንታት የጉዞ ታሪክ ያለው የቤተሰብ አባል አለ? | □ አለ □ የለም □ ካለ መቼ?____ የት?___ |

**ሠ. አካባቢያዊ አጋላጭ ነገሮች**

|  | የቤት ባለቤትነት | □ የግል □ ኪራይ □ ሌላ (ይጠቀስ) |
| --- | --- | --- |
|  | ለብቻ ማድ ቤት አለ? | □ አዎ □ የለም |
|  | የሽንትቤት አጠቃቀም ሁኔታ | □ በጋራ □ የግል □ የህዝብ |
|  | የሚጠቀሙት የሽንት ቤት ዓይነት | □ ዘመናዊ (ሴራሚክ) □ ባህላዊ (ጉድጓድ ብቻ)  □ መስክ ላይ □ ሌላ (ይጠቀስ) |
|  | የጅ መታጠቢያ በሽንት ቤት አቅራቢያ አለ? | □ አለ □ የለም |
|  | ሽንትቤቱ ምን ያክል ጊዜ ይጸዳል? | □ በሚቆሽሽ ጊዜ □ በየ ቀኑ □ ሁለት ጊዜ በሳምንት  □ አይጸዳም |
|  | ቆሻሻ ማጠራቀሚያ የሚቀመጥበት ቦታ የት ነው? | □ ግቢ ውስጥ □ ከግቢ ውጭ □ ማጠራቀሚያ የለም |
|  | የቆሻሻ ማጠራቀሚያው ተገቢ ክዳን አለው? | □ አለው □ ለውም |
|  | የቆሻሻ ማጠራቀሚያ ከሌሎት ቆሻሻውን እንዴት ያሰወግዳሉ? | □ ማቃጠል □ መቅበር □ መስክ ላይ ማፍሰስ □ ሌላ (ይጠቀስ) |
|  | ቆሻሻ አስወጋጆች በምንያክል ጊዜ ቆሻሻን ይሰበስባሉ? | □ በየቀኑ □ በየሳምንቱ □ በየወሩ □ የሚሰበስብ የለም |
|  | የቤት እንስሳት በግቢ ወስጥ አለ? | □ አለ □ የለም |
|  | ካለ ምን ዓይነት እንስሳት ነው? | □ ከብት □ ፍየል □ ውሻ □ ዶሮ □ ሌላ (ይጠቀስ) |
|  | የመጠጥ ውሃ ምንጭ | □ ቧንቧ ውሃ □ እሽግ ውሃ □ ሌላ (ትቀስ) |
|  | የውሃው ምንጭ አቅርቦት እንዴት ነው? | □ ለሰዓታት ይቋረጣል □ ለቀናት ይቋረጣል  □ ለሳምንት ይቋረጣል □ ከሳምንት በላይ ይቋረጣል  □ አይቋራረጥም |
|  | ውሃ በምን ያክማሉ? | □ ማጣራት □ ማፍላት □ የለም □ ሌላ (ይጠቀስ) |
|  | ውሃን በምን ዓይነት ዕቃ ያስቀምጣሉ? | □ ክዳን ባለው □ ክዳን በሌለው |
|  | የውሃ ማጠራቀሚ ዕቃውን በአድስ ውሃ ለመተካት ሁልጊዜ በማጠብ ባዶውን ይቀመጣል? | □ አወ □ አይደለም |

**የመረጃ ሰብሳቢ ስም _____________________ ፊርማ _________ ቀን _________**
