# Supplementary figures and images for: Molecular epidemiology and antimicrobial susceptibility of diarrheagenic Escherichia coli isolated from children under age five with and without diarrhea in Central Ethiopia
Source: PLoS One. 2023 Jul 14;18(7):e0288517. doi: 10.1371/journal.pone.0288517 (PMC10348587; doi:10.1371/journal.pone.0288517)

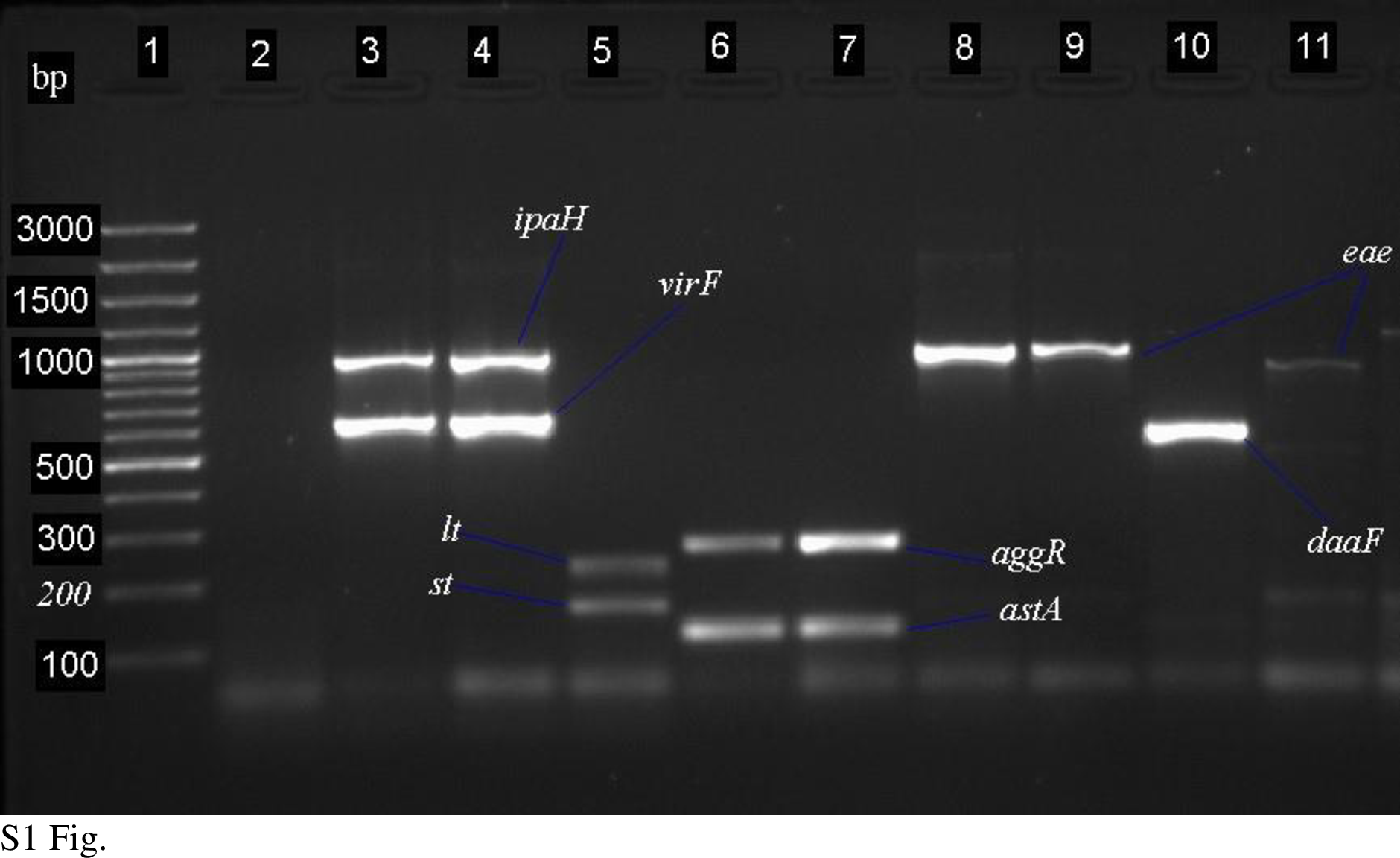

Supplement: S1 Fig — Lanes: 1 = molecular marker (GeneRuler 100 bp Plus DNA Ladder; Invitrogen Life Technologies); 2 = (negative control, CCUD 24T Escherichia coli, 3 and 4 = EIEC, 5 = ETEC, 6 and 7 = EAEC, 8, 9 and 11 = EPEC, and 10 = DAEC. Numbers on the left column denote fragment sizes (bp). (TIF) [file pone.0288517.s002.tif]

1 2 3 4 5 6 7 8 9 10 11 x

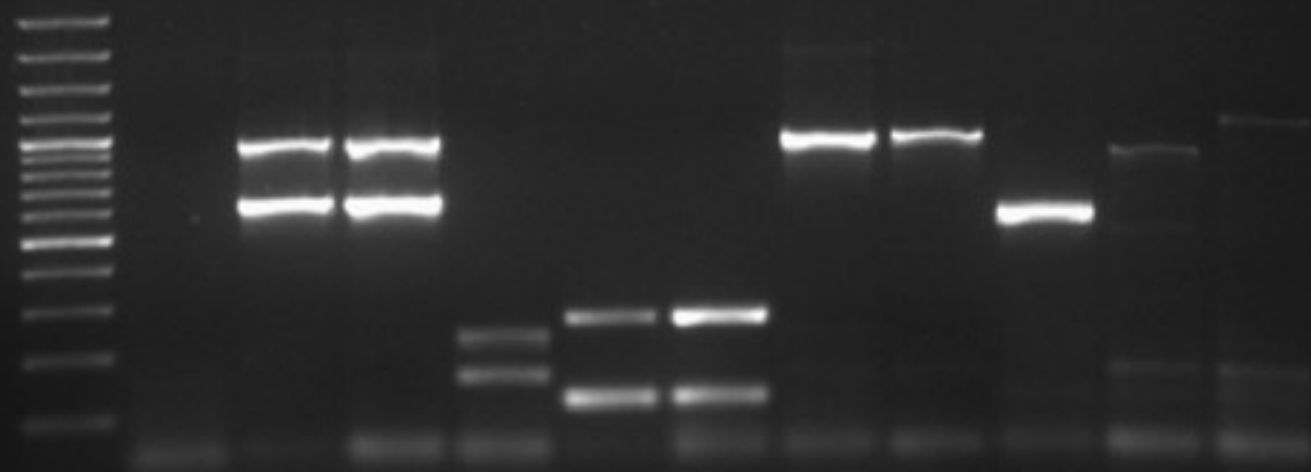

x x x x x x x x x x x x x x

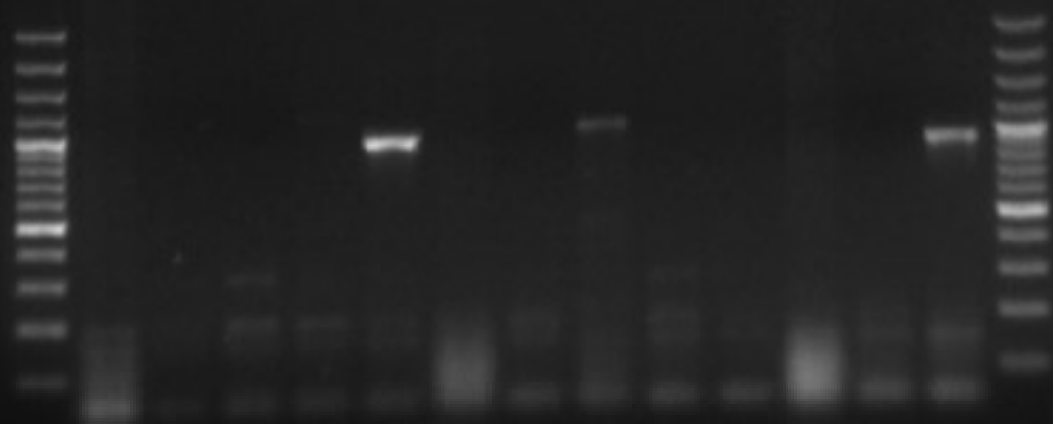

Supplement: S1 Raw images — (PDF) [file pone.0288517.s004.pdf]
