# Supplementary material for: Molecular epidemiology and antimicrobial susceptibility of diarrheagenic Escherichia coli isolated from children under age five with and without diarrhea in Central Ethiopia
Source: PLoS One. 2023 Jul 14;18(7):e0288517. doi: 10.1371/journal.pone.0288517 (PMC10348587; doi:10.1371/journal.pone.0288517)
Supplement: S1 Table — (DOCX) [file pone.0288517.s003.docx]

**S2 Table .**  Clinical presentation of under-five children those were positive for a DEC pathotype during their health-care facility visit

| Characteristics (n, %) | EPEC | ETEC | EIEC | EAEC | Mixed |
| --- | --- | --- | --- | --- | --- |
| Total (n) | 28 | 39 | 23 | 76 | 13 |
| Fever | 10 (35.7) | 11 (28.2) | 7 (30.4) | 30 (39.5) | 3 (23.1) |
| Vomiting | 12 (42.9) | 11 (28.2) | 10 (43.5) | 23 (30.2) | 6 (46.2) |
| Nausea | 6 (21.4) | 1 (2.6) | 1 (4.3) | 14 (18.4) | 0 (0%) |
| Increased thirsty | 6 (21.4) | 8 (20.5) | 6 (26.1) | 23 (30.3) | 4 (30.8) |
| Abdominal pain | 5 (17.9) | 4 (10.3) | 4 (17.4) | 8 (10.5) | 0 (0) |
| Time of illness | | | | | |
| 1-2 days | 15 (53.6) | 22 (56.4) | 16 (69.6) | 40 (52.6) | 7 (53.9) |
| 3 days | 7 (25) | 14 (35.9) | 4 (17.4) | 25 (32.9) | 6 (46.2) |
| ≥4 days | 6 (21.4) | 3 (7.7) | 3 (13) | 11 (14.5) | 0 (0) |
| Stool frequency per 24 hrs. | | | | | |
| 1-2 times | 12 (42.8) | 7 ((18) | 8 (34.8) | 19 (25) | 3 (23.1) |
| 3 times | 10 (35.7) | 18 (46.2) | 8 (34.8) | 21 (27.6) | 6 (46.2) |
| ≥4times | 21.4) | 14 (35.9) | 7 (30.4) | 36 (47.4) | 4 (30.8) |
| Dehydration | | | | | |
| Mild | 6 (21.4) | 7 (17.9) | 5 (21.7) | 25 (32.9) | 2 (15.4) |
| Moderate | 12 (42.9) | 12 (30.8) | 8 (34.8) | 31 (40.8) | 10 (76.9) |
| Severe | 0 (0) | 3 (7.7) | 1 (4.3) | 2 (2.6%) | 1 (7.7) |
| None | 10 (35.7) | 17 (43.6) | 9 (39.1) | 18 (23.7) | 0 (0) |
| Stool consistency | | | | | |
| Watery | 13 (46.4) | 11 (28.2) | 2 (8.7) | 29 (38.2) | 6 (46.2) |
| Mucoid | 10 (35.7) | 17 (43.6) | 7 (30.4) | 28 (36.7) | 2 (15.4) |
| Semisolid (loose) | 1 (3.6) | 2 (5.1) | 3 (13) | 5 (6.6) | 1 (7.7) |
| Bloody | 2 (7.1) | 4 (10.3) | 8 (34.8) | 9 (11.8) | 3 (23.1) |
| Formed | 2 (7.1) | 5 (12.8) | 3 (13) | 5 (6.6) | 1 (7.7) |

Key: DEC= Diarrheagenic *Escherichia coli*, EPEC=Enteropathogenic *Escherichia coli*, ETEC= Enterotoxigenic *Escherichia coli*, EIEC= Enteroinvasive *Escherichia coli*, EAEC=Enteroaggregative *Escherichia coli*, STEC= Shiga-toxin producing *Escherichia coli*, DEAE=Diffusely adherent *Escherichia coli*
