# Supplementary material for: Molecular epidemiology and antimicrobial susceptibility of diarrheagenic Escherichia coli isolated from children under age five with and without diarrhea in Central Ethiopia
Source: PLoS One. 2023 Jul 14;18(7):e0288517. doi: 10.1371/journal.pone.0288517 (PMC10348587; doi:10.1371/journal.pone.0288517)
Supplement: Attachment — Submitted filename: Reviewers report.docx [file pone.0288517.s005.docx]

**Reviewer’s report**

**Molecular epidemiology and antimicrobial susceptibility of diarrheagenic *Escherichia coli* isolated from children under-five with diarrhea and healthy controls in Central Ethiopia by Zenebe *et al*.**

The research article of Zenebe *et al*. described “Molecular epidemiology and antimicrobial susceptibility of diarrheagenic *Escherichia coli* isolated from children under-five with diarrhea and healthy controls in Central Ethiopia”. Moderately, the research work was well executed and the manuscript was well written. However, there are some fundamental observations that I have on the way data were presented and interpreted in the manuscript. There are lots of errors, misinterpretations, and misinformation of data in the article. In addition, there are lots of grammatical/typographical errors in the manuscript that require serious overhauling. My comments and observations are as listed below:

**Major Comments:-**

**1. Under the Methodology section, sub-section: “Detection of DEC virulence genes” on page 9:** The author gave only one PCR condition for the three different multiplex PCR done. Is the same PCR condition used for the three multiplex reactions? Is the annealing temperature of 60 oC used for the PCR processes all through the experiments? The author needs to clarify this and probably give additional information on the process.

**2. Under the Result section, sub-section: “Epidemiology of DEC and associated factors” on page 12:**

i. The statement – “Under-five children who visited a health-care facility during winter had a less likelihood…” seems to be ambiguous. Is the author saying that visitation of clinics during the summer periods brings about higher infection with the DEC compared to the winter, as if it is hospital-acquired? Or, is the author trying to say that there is a higher infection rate in the summer than in the winter thereby resulting in many children visiting the clinic due to DEC infections?
ii. The statement – “Children who lived in a family with a monthly income of below $36

 were more likely to acquire DEC (AOR=2.364, CI=1.354, 4.126) compared…” seems to be misrepresenting the data presented in Table 2. Family income range of $37-115(middle) actually has a higher number of children positive for DEC (74) compared to 61 recorded for family income of <$36 (low).

iii. The statement – “Children who were cared for by their mother had a lower risk of DEC (AOR=0.423, CI=0.211, 0.846) compared…” Like above, the statement seems not tally with the value presented in Table 2 where a higher number of children taken care of by their mother (98/183, 53.6%) actually came down with DEC infections.
iv. The statement – “Under-five children who began their supplementary food before 6 months old were more likely to acquire DEC (AOR=3.660, CI=1.322, 10.128) than those….” Same as above, the misinterpretation of actual data as expressed in Table 2. Children who began supplementary food at 6-12months have a higher incidence of DEC infections of 150 (82.0%).
v. The statement – “Children that lived in a compound with domestic animals had 2 times the likelihood (AOR=1.555, 1.016, 2.381) of being positive for DEC…” Same as above, misinterpretation of actual data expressed in Table 2. Children who were not having contact with domestic animals showed higher cases of DEC infections, precisely 114 (62.3%) compared to 69 (37.7%) observed for children that lived in a compound with domestic animals.

It could be observed that the author wrote this last statement correctly based on the data expressed in Table 2: **“Children whose family obtained water in shift had a greater change of acquiring DEC (AOR=1.735, OR=1.046, 2.879) compared to those with a continuous daily supply of water.”** I wondered why all other ones highlighted above were done differently as if the author interpreted the data with premeditated notions.

It could also be noted that the author used these wrongly expressed results to discuss findings from this research work under the Discussion section.

**3. Under the Result section, sub-section: “Antimicrobial resistance (AMR) profile” on page 15 and Table 4 on page 16:**

It could be observed that data for STEC and DEAE are conspicuously missing in Table 4, whereas, their values were included in the n=183 that was used to arrive at the values presented in the table. Likewise, the correctness of the values presented in the table should be properly checked. For example, why is the author not specific about the number of DEC isolates that were resistant to ampicillin by writing ≥85% on Line 1 under the sub-section?

Also in Table 4 EAEC = 76, whereas, if values for EAEC are summed up in Table 3, it is equal to 86. It seems there are lots of errors in the data presented in the manuscript.

**Other Comments:**

**ABSTRACT SECTION**

**Under methods, last line, page 2:** Consider revising the statement **“**Bacterial identification, antimicrobial susceptibility, and pathotype determination using polymerase chain reaction (PCR) were evaluated” to “Bacterial isolation, identification, antimicrobial susceptibility, and pathotype determination using polymerase chain reaction (PCR) were done”**.**

**Under results, line 7, page 2:** delete “of” before “non-diarrheic”

**INTRODUCTION SECTION**

**Paragraph 2, Last sentence, page 4:** The statement: “Perhaps state that recently mixed pathotypes have been identified that have virulence factors associated with more than one pathotype.” It seems something is missing in the statement or it needs to be revised to make better meaning. Also, I think cited reference “(7)” is missing there too.

**Paragraph 3, Line 5-7, page 4:** Same as above, the statement: “DEC pathotypes cause different outbreaks include in Germany in 2011(15) and Japan in 2016 (16), a haemolytic uremic syndrome caused by STEC, and in Nottingham, UK in 2014 due to EIEC (17)**”** needs revision.

**MATERIALS AND METHODS SECTION**

**Sub-section: Specimen collection-** third to the last line on page 7, add “isolation” after bacterial”

Sub-section: Bacterial Identifications on page 7 should be corrected to Bacterial Isolation and Identifications

**Sub-section: Detection of DEC virulence genes**, paragraph 2, line 2, page 8: the statement “Briefly, detection of DEC virulence genes were performed…” Should be corrected as: "Briefly, the detection of DEC virulence genes was performed..."

**Sub-section: Detection of DEC virulence genes**, paragraph 2, line 3, page 8: “…using the primer oligonucleotide DNA sequences…” should be corrected as “…using the primer sequences…”

**Sub-section: Detection of DEC virulence genes**, paragraph 2, last statement, page 9: the statement: “Known and whole genome sequenced positive DEC strains were used during optimization of the PCR assay.” is not clear.

**DISCUSSION SECTION**

**Paragraph 1, Line 1, page 17:**  The statement: In Ethiopia, molecular epidemiology of DEC and its resistance profile is unknown” sounds to be outrageous. What about works done by Getaneh et al., 2021; Belete et al., 2022 and so on? Even the author in his systematic review article (Zenebe et al., 2020) gave some insights into the occurrence of DEC in children in Ethiopia. The authors have even failed to give credit to any of the previous works done on E. coli/DEC in Ethiopia, maybe so as to prove the assertion made here. If so, it is grossly unacceptable/unscientific.

**Paragraph 5, Line 6, page 19:**

The statement: “EPEC classified in to typical…” should be revised as “EPEC are classified into typical...”

**Paragraph 7, Line 3-5, page 20:**  The statement: “In the present study children who visited a

 health-care facility during winter were less likely to be positive for DEC (table 2) compared to

 children who visited during summer.” Should be revised to make an intended meaning.

**Paragraph 7, Line 6, page 20:** “Mexco” should be corrected

**Paragraph 7, Line 7/8, page 20: The statement “**It has been reported that bacterial caused diarrhea….” Could be written as “It has been reported that bacterial-caused diarrhea…” Same thing with “…viral caused diarrhea…” in Line 8, could be written as “…viral-caused diarrhea…”

**Paragraph 7, Line 11, page 20: “**protective” should be corrected as “protected”

**Paragraph 8, Line 12-14, page 21:** What is the significance of the statement “DEC pathotypes isolated from diarrheic children showed significantly higher resistance to ceftazidime and cefotaxime compared to those from non-diarrheic children (p=0.010).”? It should be stated.
