# Supplementary material for: Molecular epidemiology and antimicrobial susceptibility of diarrheagenic Escherichia coli isolated from children under age five with and without diarrhea in Central Ethiopia
Source: PLoS One. 2023 Jul 14;18(7):e0288517. doi: 10.1371/journal.pone.0288517 (PMC10348587; doi:10.1371/journal.pone.0288517)
Supplement: Attachment — Submitted filename: Response to Reviewers.docx [file pone.0288517.s006.docx]

**'Response to Reviewers'**

**For all reviewers:**

We have learnt a lot from your comments and your comments make our manuscript more important than it was. And for this we kindly provide our gratitude to you for your professional supports. Below we go points to points.

**Reviewer #1: TITLE:**

**Comments:** The title is misleading as it indicates that healthy controls were used; however, the methods imply that children without diarrhea were enrolled, without referring to their health status. Having no diarrhea is not indicative of sound health, as all the children enrolled into the study were seeking care. So, the title needs to be revised to depict exactly what the study was about in context of children’s health.

**Responses:**

- Based on the comment we revised the title. ‘Molecular epidemiology and antimicrobial susceptibility of diarrheagenic *Escherichia coli* isolated from children under-five with and without diarrhea in Central Ethiopia’
- Some DEC pathotypes could be detected from non-diarrheic children and to see this we take non-diarrheic children who came with other medical conditions. And we explained it in the method section.

**Comments:** ABSTRACT:
The abstract indicates;

- That bacterial identification, antibiotic sensitivity testing, and pathotype determination were determined with PCR however, this is not correct especially for bacterial identification and antibiotic sensitivity testing that were phenotypically determined. It’s only DEC pathotypes that were molecularly determined. Revise.

**Responses:**

- Bacterial isolation, identification, antimicrobial susceptibility, and pathotype determination using polymerase chain reaction (PCR) were done.

**Comments:** - That 476 specimens were analyzed; were this one specimen per child? How many specimens from each child were analyzed? Revise.

**Responses:**

- One specimen was taken per child and 476 specimens mean total 476 children which were included in the analysis. More explained in method section.

**Comments:** Results for all the children are grouped together, yet as per the title and/or methods, the study seems to have subgroups i.e., healthy children / children without diarrhea vs. sick children / children with diarrhea. It would be proper to first report findings per subgroup.

**Responses:**

- The study has subgroups, children with diarrhea and children without diarrhea. And for this we did separate analysis (table 3). In table 3, DEC pathotypes are presented for case, control, AA, and DB.

**Comments:** - Percentage figures with and without corresponding fractions; it would be better to consistently use percentages with their corresponding fractions.

**Responses:**

- The comment is taken; revised in the manuscript.

**Comments:** Change ‘The overall prevalence rate -------’ to ‘The overall prevalence -------’

**Responses:**

- The overall prevalence…

**Comments:** INTRODUCTION:
In the second paragraph, revise,
- ‘Diarrheagenic E. coli (DEC) is the major causes of diarrhea -----’ to ‘Diarrheagenic E. coli (DEC) is the major cause of diarrhea -----’

**Responses:**

- cause

**Comments:** - ‘EPEC is an attaching and effacing (A/E) pathogens -----’ to ‘EPEC is an attaching and effacing (A/E) pathogen -----’

**Responses:**

- pathogen

**Comments:** - The acronyms ‘tEPEC’ and ‘aEPEC’ are not defined at their first use

**Responses:**

- typical EPEC (tEPEC)…… atypical EPEC (aEPEC) (in their first use)

**Comments:** MATERIALS AND METHODS:
- In the section ‘Study area and participants’ nowhere is it mentioned that healthy control children were enrolled as depicted in the title; revise.
**Responses:**

- Under-five children **with diarrhea** who had not received antibiotics were enrolled for the study. Under-five children who were attending the health facility for **causes other than diarrhea** and without receiving antibiotics treatment during the past three weeks were enrolled as non-diarrheic children.

**Comments:** - If healthy control children were indeed recruited, the specimen collection procedure described is for only sick children; could you describe the specimen collection procedure for healthy children?
**Responses:**

- Stool samples were taken with the same procedure from diarrheic and non-diarrheic children.

**Comments:** - ESBLs, and so are several acronyms in this manuscript, are introduced without definition at first use.
**Responses:**

Corrected at their first use

- extended spectrum ß-lactamase (ESBLs)
- polymerase chain reaction (PCR)
- deoxyribonucleic acid
- ATCC (American Type Culture Collection)
- SPSS (Statistical Package for Social Sciences)
- CCUD (Culture Collection University of Gothenburg)
- EDTA (Ethylenediamine tetraacetic acid)

**Comments:** - As well, ESBLs and carbapenemases are introduced without context and/or rationale, and the procedure for their detection is inadequate.

**Responses:**

- We included a statement in the introduction part:
  - Among MDR strains extended spectrum ß-lactamase (ESBLs) and carbapenemase producing DEC pathotypes are emerging globally (20). Nowadays, there is a serious threat due to ESBL-producing Enterobacteriaceae and an urgent threat due to Carbapenem-resistant *E. coli* [21].
- Both tests were done based on the CLSI guideline (we added the following).
- Briefly, 1μL loopful colony of test isolate from overnight blood agar plate suspended in 2 mL of nutrient broth (Oxoid UK), and 10μg of meropenem disk immersed to the nutrient broth (fully imersion). The tubes were incubated at 37°C in ambient air without agitation for 4 h ± 15 min. Subsequently, the meropenem disks were removed from the broth using an innoculaton loop. Then, the disks were placed on Mueller-Hinton agar plates (Oxoid, UK) freshly inoculated with a 0.5 McFarland suspension of Escherichia coli ATCC 25922.

**Comments:** RESULTS:
- Review presentation for the results of the ESBL- and carbapenemase producing isolates; it would be less confusing if the data for ESBL- and carbapenemase- producing isolates are presented first before showing comparisons with DEC and antibiotic sensitivity data.

**Responses:**

- Explained in result section

**Comments:** - Nothing is mentioned about carbapenemase producing isolates yet the methods are described. Revised.

**Responses:**

- mentioned in the introduction (see above response)

**Reviewer #2: Reviewer’s report**

Molecular epidemiology and antimicrobial susceptibility of diarrheagenic Escherichia coli isolated from children under-five with diarrhea and healthy controls in Central Ethiopia by Zenebe et al.

The research article of Zenebe et al. described “Molecular epidemiology and antimicrobial susceptibility of diarrheagenic Escherichia coli isolated from children under-five with diarrhea and healthy controls in Central Ethiopia”. Moderately, the research work was well executed and the manuscript was well written. However, there are some fundamental observations that I have on the way data were presented and interpreted in the manuscript. There are lots of errors, misinterpretations, and misinformation of data in the article. In addition, there are lots of grammatical/typographical errors in the manuscript that require serious overhauling. My comments and observations are as listed below:

**Comments:** Major Comments:-

1. Under the Methodology section, sub-section: “Detection of DEC virulence genes” on page 9: The author gave only one PCR condition for the three different multiplex PCR done. Is the same PCR condition used for the three multiplex reactions? Is the annealing temperature of 60 oC used for the PCR processes all through the experiments? The author needs to clarify this and probably give additional information on the process.

**Responses:**

Determination of the DEC pathotypes by PCR was done following a previous procedure with little modification after optimization. During optimization different annealing temperatures (using gradient PCR) were evaluated. For all targets at 60°C annealing temperature was optimal (the same to the previous study). The three PCR reactions were set based on the base pair sizes of the target genes (size difference) according to the manufacturer’s recommendation (amplicol size range). And we set the following PCR reactions, named M-1 to M-3 (mix).

- M-1: *bfp* (326bp), *eae* (917bp) and *stx* (518bp)
  - Base pair difference ≥192bp
- M-2: *lt* (218bp), *st* (147bp), *virF* (618bp) and *ipaH* (993bp)
  - Base pair difference ≥71bp
- M-3: *aggR* (254bp), *astA*(111bp) and *daaF* (542bp).
  - ‘Base pair difference ≥143bp

**Comments:** 2. Under the Result section, sub-section: “Epidemiology of DEC and associated factors” on page 12:

i. The statement – “Under-five children who visited a health-care facility during winter had a less likelihood…” seems to be ambiguous. Is the author saying that visitation of clinics during the summer periods brings about higher infection with the DEC compared to the winter, as if it is hospital-acquired? Or, is the author trying to say that there is a higher infection rate in the summer than in the winter thereby resulting in many children visiting the clinic due to DEC infections?

**Responses:**

- Under-five children were more likely to acquire DEC during the rainy season compared to dry season (AOR=0.529, CI= 0.335; 0.835)

**Comments:** ii. The statement – “Children who lived in a family with a monthly income of below $36
were more likely to acquire DEC (AOR=2.364, CI=1.354, 4.126) compared…” seems to be misrepresenting the data presented in Table 2. Family income range of $37-115(middle) actually has a higher number of children positive for DEC (74) compared to 61 recorded for family income of <$36 (low).

**Responses:**

- All the results presented in table 2 are based on logistic regression analysis by SPSS. Bivariate and multivariate analyses were done using a binary logistic regression model to identify determinants of DEC acquisition among under-five children. Independent variables for the final model (multivariate logistic regression) were identified using a bivariate logistic regression model with p<0.25. (we included the path used for the analysis, see the revised manuscript)
- Yes, in the actual observation (table2), the value for low family income (<$36) is lower (61) than the value for middle income ($37-115) (74). In the logistic regression analysis done for this study, we use the last variable as reference, in this case high family income ((> $115) and the interpretation was done with this reference. The recorded value may not be necessarily an indicative of statistical value. The actual occurrence of an event (DEC) could be look in terms of odd value.
- Like for example, calculating the odd value for each (low, middle, and high for DEC positive or negative), it will show the likely occurrence of the event (DEC positive or negative in this case). Odd is the probability of an event occurring divided by the probability of an event not occurring. The higher odd value for an event, is the less likely to occur (vice versa).
- Based on the above consideration the occurrence of DEC positive in low income is high (low odd value) compare to middle or high income (high odd value). (Odd for DEC positive versus negative). The odd ratio was computed by logistic regression. For more, see responses to v and below.

**Comments:** iii. The statement – “Children who were cared for by their mother had a lower risk of DEC (AOR=0.423, CI=0.211, 0.846) compared…” Like above, the statement seems not tally with the value presented in Table 2 where a higher number of children taken care of by their mother (98/183, 53.6%) actually came down with DEC infections.

**Responses:**

- The same explanation made above holds true for this. But in this case, the reference is others (grandmother, close family member, and day care). In compared to others, mother care is protective.

**Comments:** iv. The statement – “Under-five children who began their supplementary food before 6 months old were more likely to acquire DEC (AOR=3.660, CI=1.322, 10.128) than those….” Same as above, the misinterpretation of actual data as expressed in Table 2. Children who began supplementary food at 6-12months have a higher incidence of DEC infections of 150 (82.0%).

**Responses:**

- The same to the above.

**Comments:** v. The statement – “Children that lived in a compound with domestic animals had 2 times the likelihood (AOR=1.555, 1.016, 2.381) of being positive for DEC…” Same as above, misinterpretation of actual data expressed in Table 2. Children who were not having contact with domestic animals showed higher cases of DEC infections, precisely 114 (62.3%) compared to 69 (37.7%) observed for children that lived in a compound with domestic animals.

**Responses:**

- The odd for DEC positive with animal is (69/156=0.442); the odd for DEC positive with no animals is (114/137=0.832). The lower the odd is more likely to occur. The odd ratio was computed with logistic regression.

**Comments:** It could be observed that the author wrote this last statement correctly based on the data expressed in Table 2: “Children whose family obtained water in shift had a greater change of acquiring DEC (AOR=1.735, OR=1.046, 2.879) compared to those with a continuous daily supply of water.” I wondered why all other ones highlighted above were done differently as if the author interpreted the data with premeditated notions.

**Responses:** The odd for DEC positive with shift is (107/198=0.540); the odd for DEC positive with daily is (76/95=0.800). ). The lower the odd is more likely to occur. Similar with the above all results.

It could also be noted that the author used these wrongly expressed results to discuss findings from this research work under the Discussion section.

**Comments:** 3. Under the Result section, sub-section: “Antimicrobial resistance (AMR) profile” on page 15 and Table 4 on page 16:
It could be observed that data for STEC and DEAE are conspicuously missing in Table 4, whereas, their values were included in the n=183 that was used to arrive at the values presented in the table. Likewise, the correctness of the values presented in the table should be properly checked. For example, why is the author not specific about the number of DEC isolates that were resistant to ampicillin by writing ≥85% on Line 1 under the sub-section?
Also in Table 4 EAEC = 76, whereas, if values for EAEC are summed up in Table 3, it is equal to 86. It seems there are lots of errors in the data presented in the manuscript.
**Responses:**

- Instead of writing ≥85%, we revised it as ‘ampicillin (95.1%, 174/183) and tetracycline (91.3%, 167/183)
- We revised STEC and DEAE in the table under ‘others’
- In table 3, values for EAEC were incorrect in only column 9 (total calculation error) but correct in other columns. And we corrected it.

**Comments:** Other Comments:
ABSTRACT SECTION
Under methods, last line, page 2: Consider revising the statement “Bacterial identification, antimicrobial susceptibility, and pathotype determination using polymerase chain reaction (PCR) were evaluated” to “Bacterial isolation, identification, antimicrobial susceptibility, and pathotype determination using polymerase chain reaction (PCR) were done”.
Under results, line 7, page 2: delete “of” before “non-diarrheic”
**Responses:**

- Bacterial isolation, identification, antimicrobial susceptibility, and pathotype determination using polymerase chain reaction (PCR) were done
- in non-diarrheic children

**Comments:** INTRODUCTION SECTION
Paragraph 2, Last sentence, page 4: The statement: “Perhaps state that recently mixed pathotypes have been identified that have virulence factors associated with more than one pathotype.” It seems something is missing in the statement or it needs to be revised to make better meaning. Also, I think cited reference “(7)” is missing there too.

**Responses:**

- Recently mixed pathotypes have been identified that have virulence factors associated with more than one pathotypes (7).

**Comments:** Paragraph 3, Line 5-7, page 4: Same as above, the statement: “DEC pathotypes cause different outbreaks include in Germany in 2011(15) and Japan in 2016 (16), a haemolytic uremic syndrome caused by STEC, and in Nottingham, UK in 2014 due to EIEC (17)” needs revision.
**Response:**

- Due to different DEC pathotypes outbreaks were occurred in Germany in 2011(15), in Japan in 2016 (16), and in Nottingham, UK in 2014 (17).

**Comments:** MATERIALS AND METHODS SECTION
Sub-section: Specimen collection- third to the last line on page 7, add “isolation” after bacterial”

**Responses:**

- isolation,

**Comments:** Sub-section: Bacterial Identifications on page 7 should be corrected to Bacterial Isolation and Identifications

**Responses:**

- bacterial Isolation and Identifications

**Comments:** Sub-section: Detection of DEC virulence genes, paragraph 2, line 2, page 8: the statement “Briefly, detection of DEC virulence genes were performed…” Should be corrected as: "Briefly, the detection of DEC virulence genes was performed..."

**Responses:**

- the detection of DEC virulence genes was

**Comments:** Sub-section: Detection of DEC virulence genes, paragraph 2, line 3, page 8: “…using the primer oligonucleotide DNA sequences…” should be corrected as “…using the primer sequences…”

**Responses:**

- using the primer sequences

**Comments:** Sub-section: Detection of DEC virulence genes, paragraph 2, last statement, page 9: the statement: “Known and whole genome sequenced positive DEC strains were used during optimization of the PCR assay.” is not clear.
**Responses:**

- Previously used positive known DEC strains (whole genome sequenced) were used for optimization of the PCR assay.

**Comments:** DISCUSSION SECTION

Paragraph 1, Line 1, page 17: The statement: In Ethiopia, molecular epidemiology of DEC and its resistance profile is unknown” sounds to be outrageous. What about works done by Getaneh et al., 2021; Belete et al., 2022 and so on? Even the author in his systematic review article (Zenebe et al., 2020) gave some insights into the occurrence of DEC in children in Ethiopia. The authors have even failed to give credit to any of the previous works done on E. coli/DEC in Ethiopia, maybe so as to prove the assertion made here. If so, it is grossly unacceptable/unscientific.

**Responses:**

- We used ‘not well characterized’ in place of unknown. We took the comments.

**Comments:** Paragraph 5, Line 6, page 19: The statement: “EPEC classified in to typical…” should be revised as “EPEC are classified into typical...”

**Responses:**

- EPEC are…

**Comments:** Paragraph 7, Line 3-5, page 20: The statement: “In the present study children who visited a
health-care facility during winter were less likely to be positive for DEC (table 2) compared to
children who visited during summer.” Should be revised to make an intended meaning.

**Responses:**

- Under-five children were more likely to acquire DEC during the summer compared to the winter in the present study, resulting in many children visiting the health facility in the summer due to DEC infections.

**Comments:** Paragraph 7, Line 6, page 20: “Mexco” should be corrected

**Responses:**

- Mexico

**Comments:** Paragraph 7, Line 7/8, page 20: The statement “It has been reported that bacterial caused diarrhea….” Could be written as “It has been reported that bacterial-caused diarrhea…” Same thing with “…viral caused diarrhea…” in Line 8, could be written as “…viral-caused diarrhea…”

**Responses:**

- It has been reported that bacterial-caused diarrhea was high during summer and viral-caused diarrhea…

**Comments:** Paragraph 7, Line 11, page 20: “protective” should be corrected as “protected”

**Responses:**

- protected

**Comments:** Paragraph 8, Line 12-14, page 21: What is the significance of the statement “DEC pathotypes isolated from diarrheic children showed significantly higher resistance to ceftazidime and cefotaxime compared to those from non-diarrheic children (p=0.010).”? It should be stated.

**Responses:**

- we revised it.
- The finding of the present study could be explained by the fact that gut inflammation like inflammatory diarrhea can boost horizontal gene transfer between pathogenic and commensal enterobacteriaceae [55]. However, the comparative groups used in the present study were small compared to cases and it may need further matched case control study for concluding that ESBL producing DEC pathotypes are more prevalent in diarrheic than non-diarrheic children in Ethiopia.
