# Supplementary material for: Molecular epidemiology and antimicrobial susceptibility of diarrheagenic Escherichia coli isolated from children under age five with and without diarrhea in Central Ethiopia
Source: PLoS One. 2023 Jul 14;18(7):e0288517. doi: 10.1371/journal.pone.0288517 (PMC10348587; doi:10.1371/journal.pone.0288517)
Supplement: Attachment — Submitted filename: Response to Reviewers.docx [file pone.0288517.s008.docx]

**'Response to Reviewers'**

**For all reviewers:**

We have learnt a lot from your comments and your comments make our manuscript more important than it was. Your additional comment further improves the manuscript. And for this we kindly provide our gratitude to you for your professional supports. Below we go points to points.

**Reviewer’s report on the Revised Manuscript: PONE-D-22-27001R1**

**Molecular epidemiology and antimicrobial susceptibility of diarrheagenic *Escherichia coli* isolated from children under-five with diarrhea and healthy controls in Central Ethiopia by Zenebe *et al*.**

**Comments:**

There is no doubt that the quality of the manuscript has increased considerably following the revisions made by the authors. However, I have some minor observations, comments, and inputs.

1. I would like to use one of my initial comments and the authors’ response to point out my observation below:

“Comments: v. The statement – “Children that lived in a compound with domestic animals had 2 times the likelihood (AOR=1.555, 1.016, 2.381) of being positive for DEC…” Same as above, misinterpretation of actual data expressed in Table 2. Children who were not having contact with domestic animals showed higher cases of DEC infections, precisely 114 (62.3%) compared to 69 (37.7%) observed for children that lived in a compound with domestic animals.

 Responses:

 • The odd for DEC positive with animal is (69/156=0.442); the odd for DEC positive with no animals is (114/137=0.832). The lower the odd is more likely to occur. The odd ratio was computed with logistic regression.”

My observation/question is, what is the relevance of Table 3 if the data presented in it does not give direct support to the information expressed in the manuscript except an additional calculation is done by the reader of the manuscript in order to understand claims by the authors? For example, in the above comment and response, the authors have to manually calculate another set of odd values (69/156=0.442 and 114/137=0.832) to explain their point that “Children that lived in a compound with domestic animals had 2 times the likelihood (AOR=1.555, 1.016, 2.381) of being positive for DEC…” I think the authors should be able to use the data expressed in the table to write their results and discuss them in a manner that ordinary readers could understand without having to pick calculator to start doing additional calculations.

**Responses:**

1. Based on the comments given by the reviewer we revised it. We used the data (the season, child care, and availability of animal) expressed in the table **in the discussion** and make more understandable.

**Comments:**

2. Under the Introduction Section:

i. On Page 4, the revised last statement in paragraph 2, “Recently mixed pathotypes have been identified that have virulence factors associated with more than one pathotype (7).” I am surprised the citation (7) is dated 2012 whereas the authors made the statement “Recently…|”
ii. On Page 5, paragraph 4, line 4, I guessed the word “systemic” is supposed to be “systematic”. The authors should check it up. Also, the word: “presences” should be corrected to “presence” in the last statement of the same paragraph

**Responses:**

i. The reference (7), and [49] were misplaced and we corrected them. In place of reference (7) it was reference [8] which is recent data (of 2020).
ii. We corrected the systemic to systematic; presences to presence.

**Comments:**

3. Under the Methodology Section:

i. Sub-section: Phenotypic detection of ESBLs and Carbapenemases production, line 7, page 8. The statement “Briefly, 1μL loopful colony of test isolate from overnight blood agar plate suspended…” should be revised. I can’t figure out how a 1μL loopful colony of test isolate could be taken from an agar medium.
ii. Sub-section: Detection of DEC virulence genes, second to the last statement on page 10. The statement: “Previously used positive known DEC strains (whole genome sequenced) were used for the optimization of the PCR assay.” should be revised for better meaning. Previously used positive strains by who?

**Responses:**

i. We rewrite the statement using the phrase ‘a loopful (by using a 1μL size loop) of the colony of the test isolates’
ii. Based on the comment we revised the statement (highlighted in the manuscript).

**Comments:**

Additional Editor Comments :

The authors should pay close attention to the provided methodology details, results and interpretation in addition to that indicated by the reviewer. A thoroughly corrected version is desired.

Responses:

- The reference (7), and [49] were misplaced and we corrected them. We reviewed the reference list and corrected accordingly. We included DOI number for these papers that have DOI number.
- We reviewed thoroughly the paper and made all the corrections.
