# Supplementary material for: Molecular epidemiology and antimicrobial susceptibility of diarrheagenic Escherichia coli isolated from children under age five with and without diarrhea in Central Ethiopia
Source: PLoS One. 2023 Jul 14;18(7):e0288517. doi: 10.1371/journal.pone.0288517 (PMC10348587; doi:10.1371/journal.pone.0288517)
Supplement: Attachment — Submitted filename: Plosone_Major_Comments.docx [file pone.0288517.s009.docx]

Major Comments:

The present study was carried out in Ethiopia and showed the prevalence of DEC pathotypes in children under the age of five years. The authors were trying to find out and reported the prevalence of different strains of DEC pathotypes such as EAEC, ETEC, EIEC, and some hybrid strains also. The authors reported the prevalence of MDR bacteria and ESBL pathogens.

1. Diarrheagenic Escherichia coli (DEC) Pl. italicized the *Escherichia coli*.
2. Diarrheagenic Escherichia coli (DEC) is the major cause of diarrhea in under-five children. Pl. revised the sentences carefully.
3. The present study aims to determine molecular epidemiology and antimicrobial resistance profiles of DEC and identify contributing factors for acquisition among under-five children in Central Ethiopia. What is the mean of under-five children? An author should write it like children under the age of five.
4. Ethiopia is one of the 15 pneumonia and diarrhea are high-burden countries. The author should Pl. revised the sentence.
5. A systemic review and meta-analysis study done in Ethiopia reported a 22% pooled prevalence of diarrhea among under-five children. What do you mean? The author should kindly revise the sentence.
6. A systemic review and meta-analysis study done in Ethiopia reported a 22% pooled prevalence of diarrhea among under-five children. What are under five children? The author should rephrase it.
7. Specific combinations of virulence traits group DEC into six common pathotypes, including enterotoxigenic E. coli (ETEC), enteropathogenic E. coli (EPEC), Shiga-toxin producing E. coli (STEC)/enterohemorrhagic E. coli (EHEC), enteroinvasive E. coli (EIEC), enteroaggregative E. coli (EAEC), and diffusely adherent E. coli (DAEC). The following sentence is not clear. Pl. revised the sentence.
8. EPEC is an attaching and effacing (A/E) pathogen known by the A/E lesion formation. Pl. revised the sentence.
9. Based on the *E. coli* adherence factor (EAF) plasmid (pEAF) encodes bundle-forming pili (BFP). Pl. revise the sentence properly.
10. EPEC are classified into typical EPEC (tEPEC) (presence of BFP) and atypical EPEC (aEPEC). What do you mean? Pl. revised the manuscript.
11. STEC causes mild to bloody diarrhea, foodborne outbreaks, and severe illness, including hemorrhagic colitis and hemolytic uremic syndrome, and is known by its potent toxin production, Shiga toxin 1 or 2 genes (stx1, stx2). The author should rephrase the sentence and make it clear and comprehensible.
12. Transcription activator (*virF*). What is the role of virF?
13. Hybrid DEC pathotype strains are increasingly reported elsewhere in the world. What does it mean? Is it elsewhere???
14. These include EPEC/ETEC in India [10], STEC/EAEC in Germany [11] and Indonesia [12], EPEC/STEC [13] and EPEC/EAEC [14] in Brazil, and STEC/ETEC in Sweden [15]. What does it mean? Is EPEC strain also contain ETEC virulence factor?
15. Outbreaks of diarrheal diseases due to different DEC pathotypes had been reported in different countries including Germany in 2011[16], Japan in 2016 [17], and Nottingham, UK in 2014 [18]. Are any recent outbreaks of diarrhea due to DEC pathotypes like in 2018-2022?
16. Among MDR strains extended spectrum ß-lactamase (ESBLs) and carbapenemase-producing DEC pathotypes are emerging globally (20). Typological error. Pl. correct it.
17. The occurrence of hybrid DEC [10-13], outbreak-causing DEC [16-18], and resistant DEC [20, 21] reported so far showed the possible emergence of hyper-virulent DEC strains anywhere to cause severe diseases or outbreaks. The author should Pl. revised the sentence and make it easy to read and understand.
18. Epidemiological data of DEC will assist public health personnel in identifying and tracking bacterial outbreaks and severe diarrheal diseases. Thus, understanding the distribution pattern of enteric pathogens within the community will help control or prevent fatal outbreaks. Authors suddenly jumped from different strains of DAE to epidemiological factors.
19. The author should submit the structured questionnaire as the supplementary file which was used for the study.
20. How Author has prevented the contamination of urine during stool sample collection?
21. Which type of sample container was used for the stool sample collection? Pl. describe.
22. The author should briefly describe how aseptic conditions were maintained during stool sample collection.
23. A large number of typological errors were present throughout the manuscript.
24. How stool samples were inoculated on MacConkey agar?
25. Stool samples were diluted or directly streaked on MacConkey agar?
26. The author should briefly describe the inoculation of stool samples on MacConkey agar.
27. 16% glycerol stock was used?
28. Why author should store the *E. coli* isolates in Brain Heart Infusion agar? Generally, this agar is used for the cultivation of *streptococci, meningococci,* etc.
29. Ampicillin concentration was 10mcg?
30. Imipenem concentration was 30 mcg?
31. MDR was defined when the isolate was nonsusceptible to at least one agent among three or more antimicrobial categories. Is this the definition of MDR bacteria? The author should refer to the following paper for reference.
a. Singh AK, Das S, Kumar S, Gajamer VR, Najar IN, Lepcha YD, Tiwari HK, Singh S. Distribution of antibiotic-resistant Enterobacteriaceae pathogens in potable spring water of eastern Indian Himalayas: emphasis on virulence gene and antibiotic resistance genes in Escherichia coli. Frontiers in Microbiology. 2020 Nov 5;11:581072.
b. Singh AK, Das S, Singh S, Gajamer VR, Pradhan N, Lepcha YD, Tiwari HK. Prevalence of antibiotic resistance in commensal Escherichia coli among the children in rural hill communities of Northeast India. PloS one. 2018 Jun 18;13(6):e0199179.
32. What is the positive and negative control used for the Antibiotic Susceptibility test/
33. What are the inclusion and exclusion criteria used for the present study?
34. What are the basic criteria used for the selection of antibiotics?
35. by using a 1μL size loop? What is the standard size of the inoculation loop? Is it 1 μL?
36. A confirmatory test for ESBLs activity was conducted for isolates resistant to cefotaxime (30μg) and ceftazidime (30μg) by the combination disk method. The author should briefly describe the screening test method and antibiotics used in the confirmatory test.
37. *E. coli* resistance to ceftazidime and cefotaxime were considered ESBL producers.
38. What is the role of clavulanic acid during ESBL detection?
39. Without using clavulanic acid author confirmed the ESBL pathogens.
40. Subsequently, the meropenem disks were removed from the broth using an inoculaton loop. Inoculaton????
41. DEC strains positive for the relevant virulence gene (s) stored in the Armauer Hansen Research Institute from previous works and CCUD (Culture Collection University of Gothenburg) 24T E. coli were used as the positive and negative control, respectively. What is 24T E. coli? The author should describe what is the positive and negative control.
42. An author should briefly and separately describe the number of samples collected both diarrheal as well as non-diarrheal during the dry season and rainy season.
43. All 428 E. coli isolates were tested by PCR, of which 183 (42.8%, 183/428) were positive for DEC pathotype. The overall prevalence of DEC in the present study was 38.4% (183/476), and 58.5% (107/183) of the DEC types were isolated from Addis Ababa and the remaining from Debre Berhan. The line shows contradictory results to each other. The overall prevalence of DEC in the present study was 42.8% but again in the second line author mentioned that the overall prevalence of DEC type is 38.4%. The author should briefly revise the sentence.
44. The overall prevalence of DEC in the present study was 38.4% (183/476), and 58.5% (107/183) of the DEC types were isolated from Addis Ababa and the remaining from Debre Berhan (Table 3, Fig1 a & b). The current result and table 3 data were not clear.
45. In Table 3 what are the total cases meaning? And what is total control?
46. Dry season? What is the meaning of the dry season? The author did not mention any data on the dry season in table 2.
47. Under-five children were more likely to acquire DEC during the rainy season compared to the dry season (AOR=0.529, CI= 0.335; 0.835) (Table 2). In table 2 author mentioned the data of summer, Winter, and Spring whereas in the manuscript it was written as dry season and rainy season as well as some places' rainy season. The author should write it properly in the either rainy and dry seasons and spring, summer, and winter in tabl2 as well as the manuscript write-up.
48. The data of table 2 shows that the most prevalent season for DEC was winter (45.9%) followed by summer (32.2%) and spring (21.9%). Kindly check it.
49. Resistance to ceftazidime and cefotaxime was higher in DEC isolates obtained from diarrheic compared to non-diarrheic children (p=0.010). All the STEC and DAEC, 15% ETEC (6/39), and 22% EAEC (17/76) were resistant to ciprofloxacin. Kindly rephrase the sentence properly.
50. ETEC (5%, 2/39) and EAEC (3%, 2/76) were resistant to meropenem and ertapenem. The author should separately describe what percentage of ETEC as well as EAEC were resistant to meropenem as well as entrapenem.
51. This finding agrees with reports from Nigeria [42], and Iran [40]. The author should add the percentage DEC in diarrheic and non-diarrheic in Nigeria and Iran.
52. Only LT-positive ETEC was found in the non-diarrheic children in the present study. ST-positive ETEC strains are commonly associated with diarrhea compared to LT-positive. LT toxin is not responsible for diarrhea?
53. EAEC (41.5%) was the predominant DEC pathotype identified in the present study. What could be the plausible region?
54. The second most prevalent DEC pathotype was ETEC (21.3%) which was consistent with studies done in Nigeria [42], China [21], Sudan [38], and Iran [40]. The author should kindly add the data of these countries.
55. In table 2, the acquisition of DEC in the rainy season (32.2%) looks less than in the dry season (45.9%). It is quite confusing some tables show data for winter, spring, and summer whereas the author discusses the dry season and rainy season. The author should kindly revise it thoroughly and use either winter, summer as well as spring or dry and rainy seasons. What could be the possible reason for the prevalence of DEC pathotypes in winter as compared to the rainy season? The author should discuss it with suitable reason.
56. In table 2, the acquisition of DEC in the rainy season (32.2%) looks less than in the dry season (45.9%). However, it was statistically significant that under-five children were more likely to acquire DEC during the rainy season compared to the dry season in the present study, resulting in children visiting the health facility in the summer due to DEC infections. If children have visited the health facility center in the rainy season then why is the prevalence of DEC pathotypes shown higher in the winter season?
57. Children who were cared for by their mothers (37.5%) had statistically significant protection than those who were cared for by others (grandmothers, close family members, and daycare) (14.0%). The author should recheck the data properly. It is 37.5% or 53.6%??
58. This may be due to that the mother is more likely careful than other caregivers, potentially contributing to the incidence of DEC in the children. What could be the possible reason for the prevalence of DEC pathotypes in more who cares by mother as compared to other caregivers?
59. Any significant effect of the number of children in a particular family on the prevalence of DEC pathotype in that particular family?
60. What is the meaning of total control which showed in table 3?
61. All 428 E. coli isolates were tested by PCR, of which 183 (42.8%, 183/428) were positive for DEC pathotype. The overall prevalence of DEC in the present study was 38.4% (183/476), and 58.5% (107/183) of the DEC types were isolated from Addis Ababa and the remaining from Debre Berhan (Table 3, Fig1 a & b). The author should kindly revise it properly. It creates confusion like 42.8%% were DEC type and the next line overall prevalence was 38.4%.
62. In table 3 the DAEC prevalence was showing 14.5% whereas in the manuscript it was written as 41.5%. The author should revise the tables and write either in percentages or numbers.
63. The author should revise the manuscript thoroughly and correct the data in the table as well as the manuscript.
