# Supplementary material for: Molecular epidemiology and antimicrobial susceptibility of diarrheagenic Escherichia coli isolated from children under age five with and without diarrhea in Central Ethiopia
Source: PLoS One. 2023 Jul 14;18(7):e0288517. doi: 10.1371/journal.pone.0288517 (PMC10348587; doi:10.1371/journal.pone.0288517)
Supplement: Attachment — Submitted filename: Response to Reviewers.docx [file pone.0288517.s010.docx]

**For all academic editor and reviewer(s):**

First, we thank you for the comments. We have learnt a lot from your comments and your comments make our manuscript more important than it was. In addition, during the review process we got opportunity to improve the manuscript. Your additional comment further improves the manuscript. And for this we kindly provide our gratitude to you for your professional supports. We thank you to each comments and concerns. Below we go points to points. For some similar comments, we gave responses together by indicating the comment numbers.

Major Comments:

The present study was carried out in Ethiopia and showed the prevalence of DEC pathotypes in children under the age of five years. The authors were trying to find out and reported the prevalence of different strains of DEC pathotypes such as EAEC, ETEC, EIEC, and some hybrid strains also. The authors reported the prevalence of MDR bacteria and ESBL pathogens.

1. Diarrheagenic Escherichia coli (DEC) Pl. italicized the *Escherichia coli*.
   - Thank you for the comment. We took the comment and iltalized throughout the manuscript.
2. Diarrheagenic Escherichia coli (DEC) is the major cause of diarrhea in under-five children. Pl. revised the sentences carefully.
   - Based on the comment, we revised the sentence: Among the bacterial agents, diarrheagenic Escherichia coli (DEC) is the major causal **agent** of diarrhea in children under age five.
3. The present study aims to determine molecular epidemiology and antimicrobial resistance profiles of DEC and identify contributing factors for acquisition among under-five children in Central Ethiopia. What is the mean of under-five children? An author should write it like children under the age of five.
   - Again we thank you for the comments. In most literature (scholars) use the term ‘unde-five children’, ‘children under the age of five (the one you recommended)’ , or ‘children under age five’. After reviewing and considering your comment we replaced the ‘under-five children’ by ‘children under age five’ throught out the manuscript. In our case it refere to children with age of below five.
4. Ethiopia is one of the 15 pneumonia and diarrhea are high-burden countries. The author should Pl. revised the sentence.
   - We revised it: Ethiopia ranks fifth among the 15 pneumonia and diarrhea high-burden countries in deaths (due to pneumonia and diarrhea) of children under age five.
5. A systemic review and meta-analysis study done in Ethiopia reported a 22% pooled prevalence of diarrhea among under-five children. What do you mean? The author should kindly revise the sentence.
   - We took the comment and made revision: A systematic review and meta-analysis conducted in Ethiopia reported a 22% prevalence of diarrhea among children under age five.
6. A systemic review and meta-analysis study done in Ethiopia reported a 22% pooled prevalence of diarrhea among under-five children. What are under five children? The author should rephrase it.
   - We used the phrase’ children under age five’ instead (see response to comment 3).
7. Specific combinations of virulence traits group DEC into six common pathotypes, including enterotoxigenic E. coli (ETEC), enteropathogenic E. coli (EPEC), Shiga-toxin producing E. coli (STEC)/enterohemorrhagic E. coli (EHEC), enteroinvasive E. coli (EIEC), enteroaggregative E. coli (EAEC), and diffusely adherent E. coli (DAEC). The following sentence is not clear. Pl. revised the sentence.
8. EPEC is an attaching and effacing (A/E) pathogen known by the A/E lesion formation. Pl. revised the sentence.
9. Based on the *E. coli* adherence factor (EAF) plasmid (pEAF) encodes bundle-forming pili (BFP). Pl. revise the sentence properly.
10. EPEC are classified into typical EPEC (tEPEC) (presence of BFP) and atypical EPEC (aEPEC). What do you mean? Pl. revised the manuscript.
    - We revised it by taking comment 8, 9 & 10 above: Based on the presence or absence of E. coli adherence factor plasmid (pEAF), EPEC strains are classified into typical EPEC (tEPEC) that has the pEAF and atypical EPEC (aEPEC) that lacks the pEAF [6].  The plasmid (pEAF) carried a gene called *bfp* that encodes bundle-forming pili (BFP), an important virulence factor of the tEPEC strains.
11. STEC causes mild to bloody diarrhea, foodborne outbreaks, and severe illness, including hemorrhagic colitis and hemolytic uremic syndrome, and is known by its potent toxin production, Shiga toxin 1 or 2 genes (stx1, stx2). The author should rephrase the sentence and make it clear and comprehensible.
    - STEC is a foodborne and zoonotic pathogen, and it causes non-bloody diarrhea, bloody diarrhea, hemorrhagic colitis, and haemolytic uremic syndrome. STEC is known for its potent toxin called Shiga toxin encoded by *stx* genes (*stx1* and *stx2*).
12. Transcription activator (*virF*). What is the role of virF?
    - Thank you and we reconsidered it. We included this: The virF is a DNA-binding protein (also called master regulator) that control the expression of virulence factors (e.g. T3SS and effector proteins) by regulating virB (another transcriptional regulator) and icsA genes. VirB and IcsA (VirG) are required to the full expression of the invasion program by EIEC.
13. Hybrid DEC pathotype strains are increasingly reported elsewhere in the world. What does it mean? Is it elsewhere???
    - We made a slight modification on the sentence: Hybrid DEC pathotype strains are increasingly reported in different countries of the world.
14. These include EPEC/ETEC in India [10], STEC/EAEC in Germany [11] and Indonesia [12], EPEC/STEC [13] and EPEC/EAEC [14] in Brazil, and STEC/ETEC in Sweden [15]. What does it mean? Is EPEC strain also contain ETEC virulence factor?
    - Sorry, if we miss understood your comment? The statement is the continuation (linked) of the prevous sentence and this was to examplify reports of some countries. Hybrid strains are DEC strains that have virulence factors associated with more than one pathotype (explained in the previous paragraph). We revised the sentence: Among the hybrid strains reported include EPEC/ETEC in India [11], STEC/EAEC in Germany [12] and Indonesia [13], EPEC/STEC [14] and EPEC/EAEC [15] in Brazil, and STEC/ETEC in Sweden [16].
15. Outbreaks of diarrheal diseases due to different DEC pathotypes had been reported in different countries including Germany in 2011[16], Japan in 2016 [17], and Nottingham, UK in 2014 [18]. Are any recent outbreaks of diarrhea due to DEC pathotypes like in 2018-2022?
    - Based on your comment, we added some recent outbreak reports: South Korea in 2018, Japan in 2020, and United Kingdom in 2020. And we also added this: In addition, outbreaks due to different *E. coli* strains that occurred annually are available in the Centers for Disease Control and Prevention outbreak reports (<https://www.cdc.gov/ecoli/2022-outbreaks.html>).
16. Among MDR strains extended spectrum ß-lactamase (ESBLs) and carbapenemase-producing DEC pathotypes are emerging globally (20). Typological error. Pl. correct it.
    - We corrected it. From MDR bacterial strains, the extended-spectrum ß-lactamase (ESBL) and carbapenemase-producing DEC pathotypes are among the emerging pathogens globally..
17. The occurrence of hybrid DEC [10-13], outbreak-causing DEC [16-18], and resistant DEC [20, 21] reported so far showed the possible emergence of hyper-virulent DEC strains anywhere to cause severe diseases or outbreaks. The author should Pl. revised the sentence and make it easy to read and understand.
    - Based on the comment we made the revision. The occurrence of hybrid, outbreak-causing, and resistant DEC strains in different areas of the world could reveal the presence of a threat for the possible occurrence of DEC-caused severe diseases or outbreaks.
18. Epidemiological data of DEC will assist public health personnel in identifying and tracking bacterial outbreaks and severe diarrheal diseases. Thus, understanding the distribution pattern of enteric pathogens within the community will help control or prevent fatal outbreaks. Authors suddenly jumped from different strains of DAE to epidemiological factors.
    - This was to mean that providing such data will initiate public health personell to strengthen clinical laboaratory diagnostic capacity and establish active surveillance program there by enable identifying and tracking bacterial outbreaks and severe diarrheal diseases. Ultimately, prevent occurrence of severe or fatal outbreak or diseases. We did a slight modification on it after your comment.
19. The author should submit the structured questionnaire as the supplementary file which was used for the study.
    - We took the comment and submited it.
20. How Author has prevented the contamination of urine during stool sample collection?
21. Which type of sample container was used for the stool sample collection? Pl. describe.
22. The author should briefly describe how aseptic conditions were maintained during stool sample collection.
    - We considred the comments (20, 21 & 22) and we add further information on it. Training has been given to data collectors on the aseptic procedures of stool sample collection from children to avoid contamination of the stool samples (with urine, soil, or water). How to use the sterile collection materials (transport media, plastic wrap, and wooden sticks) and the critical steps in taking and transferring the stool samples were given during the training.
23. A large number of typological errors were present throughout the manuscript.
    - We thank you for the comments and we considered it. We reviewed the errors throughout the manuscript.
24. How stool samples were inoculated on MacConkey agar?
25. Stool samples were diluted or directly streaked on MacConkey agar?
26. The author should briefly describe the inoculation of stool samples on MacConkey agar.
    - Based on the comments (24, 25, &26), we included some describtion. Faecal suspension was prepared by taking the wooded sticks (cotton swabs) from the transport media containing the stool sample and rinsed thoroughly in 1ml of saline. For the liquid stool sample, saline was not used. Then, a loopful of faecal suspension (liquid stool sample) was inoculated onto MacConkey agar.
27. 16% glycerol stock was used?
28. Why author should store the *E. coli* isolates in Brain Heart Infusion agar? Generally, this agar is used for the cultivation of *streptococci, meningococci,* etc.
    - *Comment 27 & 28.* Using broth (tryptic soy broth or brain heart infusion broth) with glycerol can be used for preservation of bacterial isolates. In our case, *E. coli* isolates from each cultured plate were stored at -80°C in brain heart infusion broth containing 16% (v/v) glycerol until use. The broth was used as storage media.
29. Ampicillin concentration was 10mcg?
    - Yes, it was ampicillin (10 µg)
30. Imipenem concentration was 30 mcg?
    - No, it was meropenem (10 µg). And we made the correction.
31. MDR was defined when the isolate was nonsusceptible to at least one agent among three or more antimicrobial categories. Is this the definition of MDR bacteria? The author should refer to the following paper for reference.
a. Singh AK, Das S, Kumar S, Gajamer VR, Najar IN, Lepcha YD, Tiwari HK, Singh S. Distribution of antibiotic-resistant Enterobacteriaceae pathogens in potable spring water of eastern Indian Himalayas: emphasis on virulence gene and antibiotic resistance genes in Escherichia coli. Frontiers in Microbiology. 2020 Nov 5;11:581072.
b. Singh AK, Das S, Singh S, Gajamer VR, Pradhan N, Lepcha YD, Tiwari HK. Prevalence of antibiotic resistance in commensal Escherichia coli among the children in rural hill communities of Northeast India. PloS one. 2018 Jun 18;13(6):e0199179.
   - We thnak you for the concerns. The MDR definiation in our manuscript is based the paper: Magiorakos AP, Srinivasan A, Carey RB, Carmeli Y, Falagas ME, Giske CG, *et al.* Multidrug-resistant, extensively drug-resistant and pandrug-resistant bacteria: an international expert proposal for interim standard definitions for acquired resistance. Clin Microbiol Infect 2012;18:268–81. doi: 10.1111/j.1469-0691.2011.03570.x. The definition in this paper and in the one recommended above almost similar. In all case, MDR defined as resitance of antibiotics (at least one) in three or more classes. In our case: resistance (non-susceptible) to at least one agent (from each class) among three or more antimicrobial categories (classes) mean resitance of antibiotics in three or more classes. We put the reference while we defined it. And for better clarity we rewrote it: MDR was defined when the isolate was non-susceptible to at least one agent (from each class) in three or more antimicrobial classes.
32. What is the positive and negative control used for the Antibiotic Susceptibility test/
    - *E. coli ATCC 25922* was used as quality control strain (susceptible or positive control) for the antimicrobial susceptibility testing (as indicated in the CLSI guideline). And *K. pneumoniae* ATCC 700603 (positive control) and *E. coli* ATCC 25922 (negative control) for ESBL production, and *K. pneumonia* ATCC BAA-1705 (positive control) and *K. pneumonia* ATCC BAA-1706 (negative control) for carbapenemase production assay were used.
33. What are the inclusion and exclusion criteria used for the present study?
    - Study participants were enrolled based on inclusion and exclusion criteria. The inclusion criteria were children under age five, who did not receive antibiotics in the past three weeks, and with diarrhea. Children with the age of five or older age, and who received antibiotics treatment were the exclusion criteria. The same inclusion criteria, except diarrheic, or those who attended the health facility for causes other than diarrhea were used to include non-diarrheic children in the study.
34. What are the basic criteria used for the selection of antibiotics?
    - Antibiotics for the study were selected based on CLSI guideline for enterobacterales. In the selection we also considered previous research and prescription trends in health facilities of Ethiopia.
35. by using a 1μL size loop? What is the standard size of the inoculation loop? Is it 1 μL?
    - Thank you for the question. There are different sized inoculation loops including 1µl and 10µl inoculation loops. We used a 1µl inoculation loop based on the CLSI guideline recommendation.
36. A confirmatory test for ESBLs activity was conducted for isolates resistant to cefotaxime (30μg) and ceftazidime (30μg) by the combination disk method. The author should briefly describe the screening test method and antibiotics used in the confirmatory test.
37. *E. coli* resistance to ceftazidime and cefotaxime were considered ESBL producers.
38. What is the role of clavulanic acid during ESBL detection?
39. Without using clavulanic acid author confirmed the ESBL pathogens.
    - We thank you for the comment 36, 37, 38 and 39. We did it based on the CLSI guideline and the procedure explained in the guideline. Based on the comment we revised it. In the combination, clavulanic acid inhibit ESBL. DEC strains that were resistant at least to cefotaxime (30µg) or ceftazidime (30µg) in the screening test were selected. The ESBL production was confirmed using the combination disk method. Briefly, the combination disk method was done on MHA by using ceftazidime (CAZ) and cefotaxime (CTX) alone and with ceftazidime + clavulanic acid (CAZ/CLA) and cefotaxime + clavulanic acid (CTX/CLA) as recommended by CLSI 2020. The increase in zone size diameter by ≥5 mm for CTX/CLA and CAZ/CLA, when compared with that CTX and CAZ alone, was confirmed as the presence of ESBL.
40. Subsequently, the meropenem disks were removed from the broth using an inoculaton loop. Inoculaton????
    - We corrected it: inoculation.
41. DEC strains positive for the relevant virulence gene (s) stored in the Armauer Hansen Research Institute from previous works and CCUD (Culture Collection University of Gothenburg) 24T E. coli were used as the positive and negative control, respectively. What is 24T E. coli? The author should describe what is the positive and negative control.
    - We used DEC pathotype strains which were whole genome sequenced and confirmed positive DEC strains (EPEC, ETEC, EIEC, EAEC, STEC, and DAEC) for the target virulence gene (s) as positive control. CCUD (Culture Collection University of Gothenburg) 24T *E. coli* which did not have the potential virulence factors of the pathogenic E. coli strains, and it was obtained from University of Gothenburg. We rewrote it. DEC pathotypes (whole genome sequenced strains) confirmed positive for the target virulence gene (s) from previous works used as a positive control. CCUD (Culture Collection University of Gothenburg) 24T *E. coli* strain confirmed negative to the target gene (s) was used as negative control.
42. An author should briefly and separately describe the number of samples collected both diarrheal as well as non-diarrheal during the dry season and rainy season.
    - During the winter and spring (the dry season), 326 samples (273 diarrheic and 53 non-diarrheic children) were collected. The remaining 150 samples (118 diarrheic and 32 non-diarrheic children) were collected in the summer (the rainy season).
43. All 428 E. coli isolates were tested by PCR, of which 183 (42.8%, 183/428) were positive for DEC pathotype. The overall prevalence of DEC in the present study was 38.4% (183/476), and 58.5% (107/183) of the DEC types were isolated from Addis Ababa and the remaining from Debre Berhan. The line shows contradictory results to each other. The overall prevalence of DEC in the present study was 42.8% but again in the second line author mentioned that the overall prevalence of DEC type is 38.4%. The author should briefly revise the sentence.
    - The total samples included in the study was 476, of which we got 183 DEC strains. That mean, the prevalence of DEC is 183/476 (38.4%). 42.8% is calculated from total E. coli isolates (428), 183/428 and it is not from the total study participants. After considering your comment, we amended the statement by deleting the the figure, (42.8%, 183/428), only 183 used to avoid the confusion. All 428 *E. coli* isolates were tested by PCR, of which 183 were positive for DEC pathotype.
44. The overall prevalence of DEC in the present study was 38.4% (183/476), and 58.5% (107/183) of the DEC types were isolated from Addis Ababa and the remaining from Debre Berhan (Table 3, Fig1 a & b). The current result and table 3 data were not clear.
    - We revised it. The overall prevalence of DEC in the present study was 38.4% (183/476). Of the total DEC pathotypes, 58.5% (107/183) were from Addis Ababa, and the remaining 41.5% (76/183) were from Debre Berhan (Table 3, Fig1 a & b). The prevalence of DEC during the winter and spring (the dry season) and the summer (the rainy season) was 38% (124/326) and 39.3% (59/150), respectively.
45. In Table 3 what are the total cases meaning? And what is total control?
    - Total case and total control were to mean total diarrheic and total non-diarrheic regardless of the study areas, respectively. We revised it now, in place of total case and total control, we used total diarrheic and total non-diarrheic, respectively.
46. Dry season? What is the meaning of the dry season? The author did not mention any data on the dry season in table 2.
    - In the present study, winter, spring and summer were included. Winter and spring are dry season whereas summer is a rainy season. We indicated it in bracket in the first use, e.g. winter and spring (dry season) to mean dry season in the present study include winter and summer. We did a slight revision based on the comments.
47. Under-five children were more likely to acquire DEC during the rainy season compared to the dry season (AOR=0.529, CI= 0.335; 0.835) (Table 2). In table 2 author mentioned the data of summer, Winter, and Spring whereas in the manuscript it was written as dry season and rainy season as well as some places' rainy season. The author should write it properly in the either rainy and dry seasons and spring, summer, and winter in tabl2 as well as the manuscript write-up.
    - We took the comment and made a revision. We used winter and summer.
48. The data of table 2 shows that the most prevalent season for DEC was winter (45.9%) followed by summer (32.2%) and spring (21.9%). Kindly check it.
    - In the table 2, the data indicated the proportion not the prevalence. This mean, of 183 DEC, 84 were in winter (84/183=45.9%), 40 in spring (40/183=21.9%), and 59 in summer (59/183=32.2%). Prevalence=DEC positive/total children under age five visit during that season. We thank you for your comments.
49. Resistance to ceftazidime and cefotaxime was higher in DEC isolates obtained from diarrheic compared to non-diarrheic children (p=0.010). All the STEC and DAEC, 15% ETEC (6/39), and 22% EAEC (17/76) were resistant to ciprofloxacin. Kindly rephrase the sentence properly.
    - DEC strains resistant to ceftazidime and cefotaxime were higher in diarrheic compared to non-diarrheic children (p=0.010). All the three STECs and 1 DAEC, 15% ETEC (6/39), and 22% EAEC (17/76) strains were resistant to ciprofloxacin.
50. ETEC (5%, 2/39) and EAEC (3%, 2/76) were resistant to meropenem and ertapenem. The author should separately describe what percentage of ETEC as well as EAEC were resistant to meropenem as well as entrapenem.
    - We took the comment. Two ETECs (5%, 2/39) and two EAECs (3%, 2/76) were resistant to both meropenem and ertapenem.
51. This finding agrees with reports from Nigeria [42], and Iran [40]. The author should add the percentage DEC in diarrheic and non-diarrheic in Nigeria and Iran.
    - We took the comment and revised it. A higher prevalence of DEC (40.7%) was detected in diarrheic compared to non-diarrheic children under age five (28.2%) in the present study (p- 0.020). This finding agrees with reports from Nigeria, 76.7% and 54.6%, and Iran, 90% and 20%, in diarrheic and non-diarrheic, respectively.
52. Only LT-positive ETEC was found in the non-diarrheic children in the present study. ST-positive ETEC strains are commonly associated with diarrhea compared to LT-positive. LT toxin is not responsible for diarrhea?
    - It is responsible. It was to mean that the ST-positive ETEC is more frequently found in severe infections compared to LT-positive. Revised accordingly.
53. EAEC (41.5%) was the predominant DEC pathotype identified in the present study. What could be the plausible region?
    - The possible reason for its predominant epidemiology may be associated with its genomic plasticity. It is relatively more genetically hetrogenetic and an emerging pathogen. The heterogeneity of EAEC could contribute for its epidemiology.
54. The second most prevalent DEC pathotype was ETEC (21.3%) which was consistent with studies done in Nigeria [42], China [21], Sudan [38], and Iran [40]. The author should kindly add the data of these countries.
    - We did the revision. The second most prevalent DEC pathotype was ETEC (21.3%) which was consistent with reports of 17.3% in Nigeria [42], 14.8% in China [21], 18% in Sudan [38], and 26% in Iran [40].
55. In table 2, the acquisition of DEC in the rainy season (32.2%) looks less than in the dry season (45.9%). It is quite confusing some tables show data for winter, spring, and summer whereas the author discusses the dry season and rainy season. The author should kindly revise it thoroughly and use either winter, summer as well as spring or dry and rainy seasons. What could be the possible reason for the prevalence of DEC pathotypes in winter as compared to the rainy season? The author should discuss it with suitable reason.
    - We took the comments to avoid the confusion. And we used winter (dry season) and summer (rainy season). 32.2% for summer (rainy season) is not a prevalence, it is a proportion (59/183, not 59/150). Actual prevalence for summer is 59/150=39.3% and for winter 84/230=36.5%. DEC is more prevalence in summer than winter and we reviewed it.
56. In table 2, the acquisition of DEC in the rainy season (32.2%) looks less than in the dry season (45.9%). However, it was statistically significant that under-five children were more likely to acquire DEC during the rainy season compared to the dry season in the present study, resulting in children visiting the health facility in the summer due to DEC infections. If children have visited the health facility center in the rainy season then why is the prevalence of DEC pathotypes shown higher in the winter season?
    - See response to comment 55. In fact, the prevalence is higher in summer compared to winter in this case.
57. Children who were cared for by their mothers (37.5%) had statistically significant protection than those who were cared for by others (grandmothers, close family members, and daycare) (14.0%). The author should recheck the data properly. It is 37.5% or 53.6%??
    - The figures are correct, we took the DEC negative in both cases. In table 2, when we look the proportion for DEC positive in children cared by mother (53.6%) is higher than others (8.2%). If we calculate the prevalence of DEC in children cared by mother (98/208=47%) and in other (15/56=27%). Still the prevalence is higher in children cared by mother than cared by other. But, this is not ststistically significant. Instead, the statistical value indicated that children who cared by mother are protected compared to children cared by other. We also tried to see the actual occurrence of an event (DEC) through odd value. Odd is the probability of an event occurring divided by the probability of an event not occurring. The higher odd value for an event, is the less likely to occur (vice versa). The odd for DEC positive with mothers is (98/110=0.891); the odd for DEC positive with other is (15/41=0.366). The lower the odd is more likely to occur. In this case, DEC more occur with other (regardless of the proportion and prevalence value). Thus, in the discussion, we used the figure of DEC negative to avoid confusion. Children who were cared for by their mothers had statistically significant protection (37.5% DEC negative) than those who were cared for by others (grandmothers, close family members, and daycare) (14.0% DEC negative).
58. This may be due to that the mother is more likely careful than other caregivers, potentially contributing to the incidence of DEC in the children. What could be the possible reason for the prevalence of DEC pathotypes in more who cares by mother as compared to other caregivers?
    - See response to 57
59. Any significant effect of the number of children in a particular family on the prevalence of DEC pathotype in that particular family?
    - Number of children in the family did not found associated with DEC acquisition in the present study (p>0.25) and not included in the multivariate logistic regression.
60. What is the meaning of total control which showed in table 3?
    - Based on your comment, we revised it (see response to comment 45).
61. All 428 E. coli isolates were tested by PCR, of which 183 (42.8%, 183/428) were positive for DEC pathotype. The overall prevalence of DEC in the present study was 38.4% (183/476), and 58.5% (107/183) of the DEC types were isolated from Addis Ababa and the remaining from Debre Berhan (Table 3, Fig1 a & b). The author should kindly revise it properly. It creates confusion like 42.8%% were DEC type and the next line overall prevalence was 38.4%.
    - 183 is from 428 total E. coli (DEC from total E.coli isolates, not from total study participants). The 428 E.coli were from 476 children. The total sample size is 476. And the prevalence of DEC among the 476 children is 183/476=38.4%. And we put the numerator and denomnnator to avoid the confusion.
62. In table 3 the DAEC prevalence was showing 14.5% whereas in the manuscript it was written as 41.5%. The author should revise the tables and write either in percentages or numbers.
    - In the present study, among the pathotypes, DAEC (0.6%, 1/183) and EAEC (41.5%, 76/183) were detected. We checked it.
63. The author should revise the manuscript thoroughly and correct the data in the table as well as the manuscript.
    - We took all the comments in to consideration and did the revision. Lastly, we provide our gratitude for your constructive comments.
