# Supplementary material for: Molecular epidemiology and antimicrobial susceptibility of diarrheagenic Escherichia coli isolated from children under age five with and without diarrhea in Central Ethiopia
Source: PLoS One. 2023 Jul 14;18(7):e0288517. doi: 10.1371/journal.pone.0288517 (PMC10348587; doi:10.1371/journal.pone.0288517)
Supplement: Attachment — Submitted filename: Minor_Comments (1).docx [file pone.0288517.s011.docx]

Q.1 Then, the disks were placed on Mueller-Hinton agar plates (Oxoid, UK) freshly inoculated with a 0.5 McFarland suspension of *Escherichia* coli ATCC 25922. The results from the ESBL confirmation and carbapenemase production were interpreted according to CLSI guidelines.

Kindly italicized the coli also.

Q.2 DEC pathotypes (whole genome sequenced strains) confirmed positive for the target virulence gene(s) from previous works used as a positive control. CCUD (Culture Collection University of Gothenburg) 24T *E. coli* strain confirmed negative to the target gene (s) was used as a negative control. Kindly correct the typological errors.

Q.3 A clinical presentation of children under age five positive for DEC is presented in S2Table. Pl. correct the typological errors.

Q.4 Of the total DEC pathotypes, 58.5% (107/183) were from Addis Ababa, and the remaining 41.5% (76/183) were from Debre Berhan (Table 3, Fig1 a & b). Table 3 or Table 2?? Pl. correct it carefully.

Q.4 Placed in a freezer at −20°C for 10 minutes. Kindly correct the typological errors.

Q.5 Then, 100μL of the supernatant was transferred into a nuclease-free Eppendorf tube and stored at −20°C until use. Kindly correct the typological errors.

Q.6 PCR reaction *3* contained primer mix 3 (M-3) for the detection of EAEC and DAEC targeting virulence genes *aggR,* *astA*, and *daaF*. Why italicized the 3? In case it has special meaning, state it, or correct it.

Q.7 The PCR thermal conditions were set with an initial denaturation of 94°C for 2 min, followed by 35 cycles of 92°C for 30 sec, annealing at 60°C for 30 sec, extension at 72°C for 30 sec, and a final extension at 72°C for 5 min in a PCR machine (Biometra TRIO Thermal Cycler, Analytik Jena). Kindly correct the typological errors.

Q.8 Table 3 and Table 4 were showing the number of isolates as well as the percentage also. It creates confusion for the readers. Kindly show the data only in percentages as it is better to understand.

Q.9 Author is showing two types of data one is the proportion of DEC pathotypes and the second one is the prevalence of DEC pathotypes. It will create confusion for the readers. Suitably change to make them comparable.

Q.10 What could be the plausible reason to collect the diarrheal stool sample from the children who did not receive the antibiotics in the past three weeks? Briefly indicate reason/logic.

Q.11 **Table 4.** Antimicrobial resistant profile of DEC pathotypes (n=183) isolated from diarrheic and non-diarrheic under -five children, Addis Ababa and Debre Berhan, Ethiopia. Kindly correct it properly.
