# Supplementary material for: Molecular epidemiology and antimicrobial susceptibility of diarrheagenic Escherichia coli isolated from children under age five with and without diarrhea in Central Ethiopia
Source: PLoS One. 2023 Jul 14;18(7):e0288517. doi: 10.1371/journal.pone.0288517 (PMC10348587; doi:10.1371/journal.pone.0288517)
Supplement: Attachment — Submitted filename: Response to Reviewers.docx [file pone.0288517.s012.docx]

**For all academic editor and reviewer(s):**

First, we thank you for the comments. We have learnt a lot from your comments and your comments make our manuscript more important than it was. In addition, during the review process we got opportunity to improve the manuscript. Your additional comment further improves the manuscript. And for this we kindly provide our gratitude to you for your professional supports. We thank you to each comments and concerns. Below we go points to points.

Minor comments

Q.1 Then, the disks were placed on Mueller-Hinton agar plates (Oxoid, UK) freshly inoculated with a 0.5 McFarland suspension of *Escherichia* coli ATCC 25922. The results from the ESBL confirmation and carbapenemase production were interpreted according to CLSI guidelines.

Kindly italicized the coli also.

- We thank you for the comment. We italiced it.

Q.2 DEC pathotypes (whole genome sequenced strains) confirmed positive for the target virulence gene(s) from previous works used as a positive control. CCUD (Culture Collection University of Gothenburg) 24T *E. coli* strain confirmed negative to the target gene (s) was used as a negative control. Kindly correct the typological errors.

- We corrected it. DEC pathotypes (whole genome sequenced strains) confirmed positive for the target virulence gene(s) from previous works used as the positive control. The CCUG (Culture Collection University of Gothenburg) 24T E. coli strain confirmed negative for the target gene (s) was used as a negative control.

Q.3 A clinical presentation of children under age five positive for DEC is presented in S2Table. Pl. correct the typological errors.

- We corrected it. The clinical presentation of children under age five positive for DEC is presented in S2 Table.

Q.4 Of the total DEC pathotypes, 58.5% (107/183) were from Addis Ababa, and the remaining 41.5% (76/183) were from Debre Berhan (Table 3, Fig1 a & b). Table 3 or Table 2?? Pl. correct it carefully.

- We corrected it. It is Table 2. (table 3 also have the data but table 2 is more clear).

Q.4 Placed in a freezer at −20°C for 10 minutes. Kindly correct the typological errors.

- We corrected it. −20 °C

Q.5 Then, 100μL of the supernatant was transferred into a nuclease-free Eppendorf tube and stored at −20°C until use. Kindly correct the typological errors.

- We corrected it. −20 °C

Q.6 PCR reaction *3* contained primer mix 3 (M-3) for the detection of EAEC and DAEC targeting virulence genes *aggR,* *astA*, and *daaF*. Why italicized the 3? In case it has special meaning, state it, or correct it.

- *aggR,* *astA*, and *daaF* are among the target genes and we italized all the genes (like *bfp, eae*, and *stx* as well as *lt*, *st*, *virF*, and *ipaH)*.

Q.7 The PCR thermal conditions were set with an initial denaturation of 94°C for 2 min, followed by 35 cycles of 92°C for 30 sec, annealing at 60°C for 30 sec, extension at 72°C for 30 sec, and a final extension at 72°C for 5 min in a PCR machine (Biometra TRIO Thermal Cycler, Analytik Jena). Kindly correct the typological errors.

- We corrected it. The PCR thermal conditions were set with an initial denaturation of 94 °C for 2 min, followed by 35 cycles of 92 °C for 30 sec, annealing at 60 °C for 30 sec, extension at 72 °C for 30 sec, and a final extension at 72 °C for 5 min in a PCR machine (Biometra TRIO Thermal Cycler, Analytik Jena).

Q.8 Table 3 and Table 4 were showing the number of isolates as well as the percentage also. It creates confusion for the readers. Kindly show the data only in percentages as it is better to understand.

- Based on the comment, we put the data in percentage for both tables.

Q.9 Author is showing two types of data one is the proportion of DEC pathotypes and the second one is the prevalence of DEC pathotypes. It will create confusion for the readers. Suitably change to make them comparable.

- Thank you for the concerns. We use proportions in all our tables. The proportion could indicate the DEC positive from the total for each variable (e.g. in table 2). We use prevalence in some cases (data in table 2) in the discusions to make more clear on the issue (to avoid confusion for the readers).

Q.10 What could be the plausible reason to collect the diarrheal stool sample from the children who did not receive the antibiotics in the past three weeks? Briefly indicate reason/logic.

- Prior antibiotic use can affect pathogen distribution [33]. Although the exact time for antibiotics to clear from the body is varied (due to many factors), some antibiotics take a short time (24hrs) [34], and others, such as azithromycin [35], take a longer time (more than two weeks). For this reason, only those who had no antibiotic exposure in the past three weeks were included in the study.

Q.11 **Table 4.** Antimicrobial resistant profile of DEC pathotypes (n=183) isolated from diarrheic and non-diarrheic under -five children, Addis Ababa and Debre Berhan, Ethiopia. Kindly correct it properly.

- We corrected it. Antimicrobial resistant profile of DEC pathotypes (n=183) isolated from diarrheic and non-diarrheic children under age five in Addis Ababa and Debre Berhan, Ethiopia.
